# Whole-Body [^18^F]FDG-PET/CT Imaging of Healthy Controls: Test/Retest Data for Systemic, Multi-Organ Analysis

**DOI:** 10.1038/s41597-025-05997-4

**Published:** 2025-10-29

**Authors:** Sebastian Gutschmayer, Josef Yu, Barbara Katharina Geist, Öykü Özer, Bettina Reiterits, Daria Ferrara, Manuel Pires, Ivo Rausch, Harald Ibeschitz, Georgios Karanikas, Lalith Kumar Shiyam Sundar, Lukas Nics, Zacharias Chalampalakis, Dina Muin, Werner Langsteger, Marcus Hacker, Thomas Beyer

**Affiliations:** 1 QIMP Team, Medical University of Vienna, Vienna, Austria; 2 Division of Nuclear Medicine, Medical University of Vienna, Vienna, Austria; 3 DIGIT-X Lab, Department of Radiology, LMU Munich, Munich, Germany

**Keywords:** Whole body imaging, Molecular imaging, Population screening, Computed tomography, Positron-emission tomography

## Abstract

Positron Emission Tomography (PET) with 2-deoxy-2-[^18^F]fluoro-D-glucose (FDG) and Computed Tomography (CT) are key tools in oncology diagnostics. However, most PET studies focus on tumour detection. We propose that a holistic assessment of the macroenvironment using FDG-PET could enhance our understanding of systemic treatment interactions and improve patient care. To achieve this, we aim to develop a normative database for FDG-PET based on healthy controls, providing a reference for identifying voxel-level metabolic aberrations in cancer patients. This approach may uncover treatment-related changes beyond cancer diagnostics. 48 healthy controls who underwent dynamic test-retest whole-body PET/CT imaging post [^18^F]FDG injection were included in this cohort. Static PET images (57–62 min post-injection) were reconstructed using CT-based attenuation and scatter correction, with iterative reconstruction incorporating resolution recovery and time-of-flight data. Standardized uptake values (SUVs), tissue densities (HU), and volumes for 135 organs were calculated using in-house segmentation software. The dataset, including anonymized PET/CT images and CT-derived segmentations in NIfTI format, supports the creation of a normative FDG-PET database and enables multi-organ analyses using PET/CT imaging.

## Background & Summary

The global incidence and prevalence of complex diseases — such as cancer, cardiovascular disorders, neurodegenerative conditions, and metabolic syndromes — have risen steadily in recent years^1–3^. Early diagnosis and the implementation of personalized treatment strategies are essential for improving patient outcomes, including survival rates and quality of life. To this end, diagnostic tools, particularly blood-based biomarkers and non-invasive imaging techniques, play a pivotal role.

Non-invasive anatomical imaging techniques, such as Computed Tomography (CT), provide high spatial resolution for visualizing patient anatomy and pathomorphology. In contrast, functional imaging methods, like Positron Emission Tomography (PET), offer molecular-level insights with high specificity through the use of targeted radiotracers. Together, these imaging modalities—CT and PET—deliver complementary visual and quantitative data that are often indispensable for guiding treatment decisions.

The integration of PET and CT into a dual-modality PET/CT system has demonstrated exceptional sensitivity and specificity in detecting and characterizing malignant diseases^4^. Moreover, PET/CT imaging has proven invaluable for enhancing diagnosis, monitoring disease progression, and predicting therapeutic responses across a broad spectrum of cancer patients^5^.

Today, over 80% of all PET scans are conducted for oncology indications, utilizing 2-deoxy-2-[^18^F]fluoro-D-glucose (FDG) as the tracer of choice. FDG highlights regions of increased glucose metabolism, a hallmark of cancer tissue. Whole-body PET (WB-PET) imaging, which scans the entire patient, is commonly employed for this purpose^6,7^. Metastatic lesions are identified on WB-PET scans by detecting abnormally elevated local FDG accumulations, visualized in three-dimensional representations of the patient’s anatomy.

This approach—relying on hypermetabolic uptake patterns in FDG-PET images—is widely used in oncology diagnostics^8^. However, it underutilizes the full potential of PET imaging. Current analytical methods often treat each organ as an isolated entity, overlooking the intricate metabolic interactions between organs^9^. This reductionist perspective is particularly limiting when assessing systemic diseases, such as chronic conditions, which disrupt metabolic homeostasis and affect multiple organs simultaneously through disease-specific networks^10^.

In addition to identifying pathological aberrations, including tumours, heart disease^11^, and neurodegenerative disorders^12^, FDG-PET imaging has also emerged as the method of choice for assessing whole-body metabolic health^13^. Prior studies of FDG uptake in patients already hinted at the value of physiological FDG uptake in normal tissues throughout the body as an “adjunct to physician experience” that comes with “the potential to improve interpretive accuracy”^14^. Today, WB- FDG-PET scans may add crucial information on “normal” tracer uptake patterns, offering a systemic perspective on inter-organ metabolic networks^9^.

Detecting associated variations in FDG accumulation across multiple organs differs fundamentally from tumour lesion-based detections^8^, and is often challenging for a single observer studying a single patient, relying solely on visual assessments. Therefore, the establishment of a normative atlas of FDG-PET information, together with the provision of automated and AI-supported methods, may assist in evaluating these systemic effects by enabling the detection of subtle metabolic deviations that precede overt disease manifestation, such as in the early stages of cachexia^15^.

Nonetheless, publicly available imaging databases remain scarce in their representation of PET data of healthy controls. The *AutoPET* challenge provides open access to 1014 whole-body FDG-PET/CT image sets. However, this database does not include true healthy controls, but FDG-PET negative scans of cancer patients^16^. Two other community efforts provide open access to PET data, but do not entail WB-PET images of healthy controls^17,18^. Still, the value and results of open-sourcing PET-based datasets can already be appreciated^19^. The *Cancer Imaging Archive* (TCIA) hosts a variety of imaging datasets, but only one such dataset has been obtained from healthy controls who underwent low-dose CT imaging alone^20^.

As part of our ENHANCE.PET initiative^21^, we curated FDG-PET/CT datasets of healthy controls (subjects) who underwent two PET/CT scans in a test-retest setting (hereafter referred to as Test and Retest) with a one-month interval between them. Each dataset (Test and Retest) includes 48 whole-body PET/CT scans following the injection of 100 MBq of FDG and a 62-minute dynamic emission scan, along with corresponding low-dose CT images acquired for the purpose of CT-based attenuation (and scatter) correction^22–24^. In addition, we provide the organ-based segmentations of 135 healthy tissues per scan, incorporating both CT and PET readouts. The segmentations were generated using our in-house tool, MOOSE version 3.0.13^25^. The dataset is provided in anonymized NIfTI format - with PET and CT images being defaced - to ensure subject privacy, along with demographic details and CT and PET acquisition parameters as non-imaging metadata.

It is understood that FDG uptake is influenced by a number of confounders^26^, including age^27,28^, body mass index^29,30^, sex^31^, ethnicity^32^, and physical activity levels of subjects^33,34^. Within our original project, we seek to build a normative atlas of glucose uptake - as measured by WB-FDG-PET - across multiple organs in Caucasian controls. In addition, we intend to study the reproducibility and repeatability of FDG-PET readouts in this healthy control cohort so as to define a baseline against which disease-induced metabolic aberrations (in similar cohorts) can be evaluated.

As detailed in the Technical Validation Section, this dataset is well-suited for establishing reliable reference values for FDG uptake in healthy controls (Caucasian, see ethnicity as a confounder). It also enables further validation of glycolytic activity across organs as a potential surrogate marker for systemic metabolic alterations induced by cancer, contingent on the analysis of confounder-matched cancer cohorts. This initiative aligns with our overarching goal of advancing healthcare by extending beyond a cancer-centric focus to offer a holistic perspective on disease diagnosis and management. By doing so, it enhances our understanding of health and disease at a broader, more systemic level.

We believe that making this standardized dataset - though limited in size - openly available will significantly contribute to the differential understanding of healthy and pathological tissues in computational medicine. By providing a comprehensive resource, we aim to facilitate future research and advanced data analysis in PET/CT imaging, particularly in areas such as multi-modal imaging analysis and the development and validation of deep learning algorithms.

## Methods

### Data collection

This dataset includes WB-FDG-PET/CT imaging data and subsequently derived CT and PET readouts for 135 organs of 48 healthy controls: 25 (52%) females and 23 (48%) males. All subjects were adults with a mean age of 38 ± 14 years (range: 19–59 y/o). The status of health, i.e., “healthy”, was defined as the known absence of systemic diseases, such as cancer or diabetes, as verified during the recruitment and initial subject consultation.

**Fig. 1:**
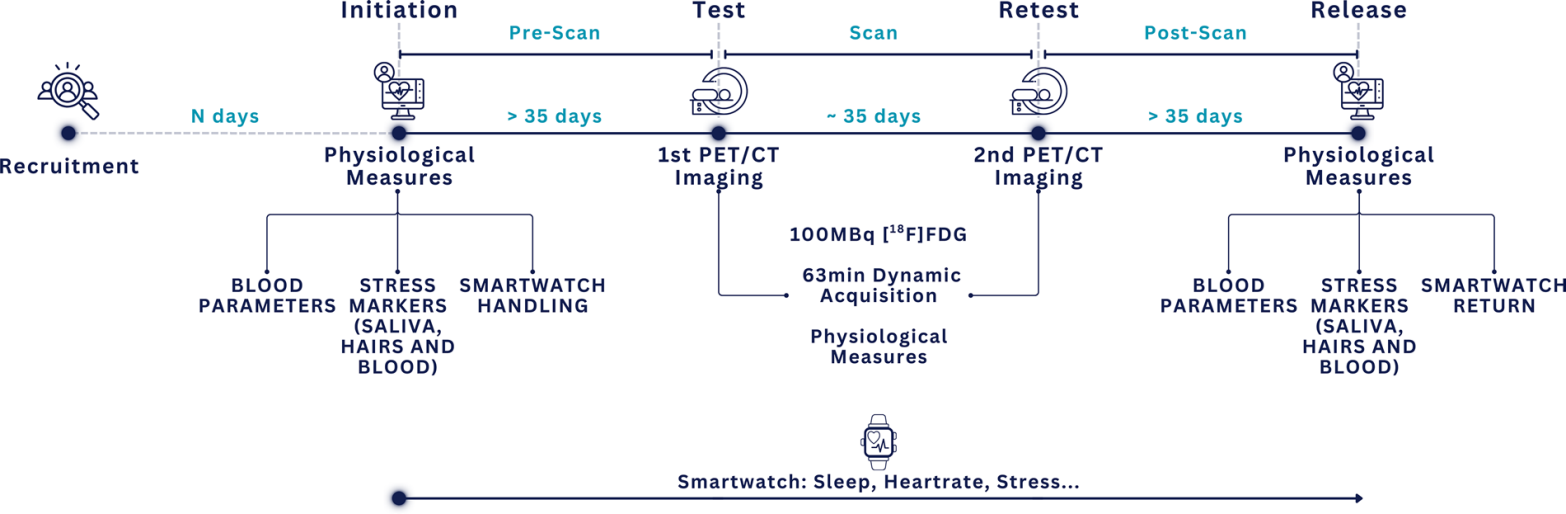
Healthy control study protocol: Recruited subjects were enrolled on day 1 for a clinical assessment, incl. blood parameters and stress markers (“Initiation”). On that day, subjects were given the smartwatch and instructed to wear it continuously throughout the entire study period. After ~5 weeks (day ~35), subjects were invited to the 1st PET/CT scan (Test) and the collection of blood and stress markers. After 4-5 weeks (day ~70), the same procedure (Retest) was repeated, and subjects were released for continuous wearable monitoring. After ~100 days, the subjects were called in for their clinical assessment, incl. blood parameters and stress markers (“Release”). Also, all subjects handed over their smartwatch, and their bio readouts were extracted and anonymized to be stored locally on-site.

All verified healthy controls were included in a 6-month study (Fig. 1). Clinical tests for stress markers (hair, blood), inflammatory and other biochemical markers (blood) were performed four times throughout the study period: on day 1, the day of the Test PET/CT scan, the day of the Retest PET/CT scan, and again on the last day of the study. The time between the Test and the Retest PET/CT was 4-5 weeks. All subjects were given a wearable device (Garmin Forerunner 955) on day 1 and asked to wear it continuously throughout the entire study period to document biosignals against which any variations in the metabolic patterns seen on the FDG-PET images of the Test and Retest scan will be compared at a later time.

**Fig. 2:**
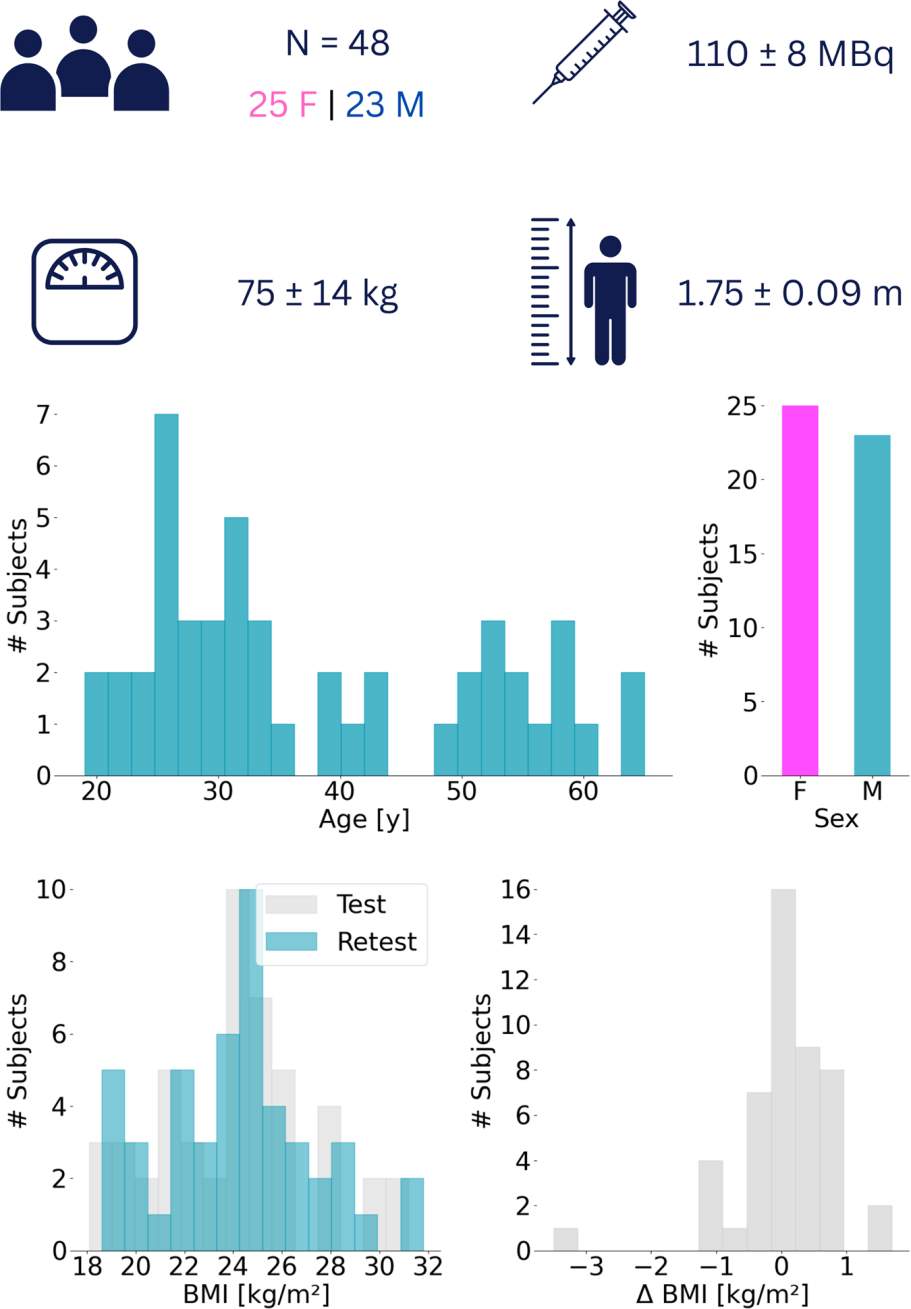
Demographics of the 48 healthy controls included in the complete study. The age, sex, BMI, and Δ BMI distributions of the 48 subjects are presented as histograms. For Δ BMI, the measurements derived from the Test scan served as a reference.

All datasets were acquired in accordance with the Declaration of Helsinki, with written informed consent obtained from all subjects prior to examinations. All data were acquired between Sep 28, 2023, and Aug 5, 2024, at the Medical University of Vienna, Austria, following IRB approval (EK 1707/2022), which can also be found in the public registry of approved studies of the IRB: https://ekmeduniwien.at/core/catalog/2023/. The demographics of the study participants – recruited via the University network and adverts in cooperating institutions - are summarized in Table S1, and an overview is presented in Fig. 2.

Of note, this study was approved for the enrollment of 50 healthy controls. After the completion of the first FDG-PET/CT scan (Test, Fig. 1), 2/50 controls presented with incidental findings indicative of malignant disease. Following the bioptic confirmation of the image-based findings, both subjects were excluded from the study and transferred to standard clinical workup.

### Imaging protocol

PET/CT imaging was conducted using a Biograph Vision QUADRA system (Siemens Healthineers, USA) with software version VR20B. This system integrates a 128-slice dual-energy CT alongside a PET component comprising four VISION PET units^35^. The system offers a 106 cm axial field of view (FOV), with a transverse and axial spatial resolution of 3.3 mm and 3.8 mm, respectively, at a 1 cm FOV offset. It achieves a sensitivity of 180 kcps/MBq with a peak NEC of 276 kcps at 25 kBq/mL. For detailed technical specifications and performance parameters, refer to^36–38^. To ensure the reliability of quantitative measurements, daily quality control procedures are performed for both PET and CT components, along with regular cross-calibration checks, conducted at least every six months and additionally following any changes to the imaging systems or dose calibrators.

Prior to the Test and Retest PET/CT scan, all subjects were asked to fast for 4 hours (blood glucose level < 150 mg/dL). Subjects were positioned head-first in a supine position with arms down and asked to lie still with the permission to breathe normally (shallow) during the duration of the examination (CT and PET for Test and Retest). A low-dose spiral CT scan of the entire imaging range with an automatic tube current modulation set at 25 mA reference and a tube voltage of 140 kVp using the zinc filter and quality setting 3 was performed first. The scanning parameters included a table feed per rotation of 38 mm and a spiral pitch factor of 1.0. Reconstruction was done using the iterative reconstruction algorithm ADMIRE and using the convolutional kernel Br32f and a reconstruction diameter of 780 mm. The reconstructed images had a pixel spacing of 1.523 mm × 1.523 mm, with a slice thickness of 2 mm. The corresponding matrix size was 512 × 512 × 531 voxels.

Following the completion of the CT scan, subjects were moved to the PET imaging position and injected with 100 MBq [^18^F]FDG. The PET emission scan was set to start immediately, and listmode emission data were acquired for 62 min. At the end of the examination, the subjects were released and asked to void. The total effective dose received by the participating subjects was estimated to be 3.0 mSv, with 33% from the LD-CT and 67% from the emission scan.

PET image data were reconstructed for 57–62 min post-injection using OP-OSEM with TOF information and PSF correction with 4 iterations and 5 subsets. A matrix size of 440 × 440 × 531 voxels with an in-plane voxel spacing of 1.65 mm × 1.65 mm and a slice thickness of 2 mm was used, and a Gaussian post-reconstruction filter of 2 mm full width at half maximum (FWHM) was applied. All standard corrections were utilized, comprising of CT-based attenuation and scatter correction, as well as corrections for randoms, decay, and dead time.

CT and PET reconstruction of all healthy subjects shared in this cohort resulted in 48 PET image volumes matched to 48 CT image sets at two time points along the study trajectory (Fig. 1: Test and Retest). Both PET/CT imaging sessions were set apart by an average of (38 ± 10) days (minimum: 22 days, maximum: 98 days, Table S1). All PET images were converted from Bq/mL to weight-based standardized uptake values (SUV). See Table S1 for details on key protocol parameters and reported scan and subject differences between Test and Retest.

### Data processing, image segmentation, and readouts

All 50 participants of this study had their PET/CT examinations reviewed by a board-certified nuclear medicine physician for image quality assessment and incidental findings. As stated, 2/50 enrolled subjects were excluded from this study after confirming their incidental findings on the Test PET/CT (Fig. 1). The acquired PET and CT images of the remaining healthy controls (n = 48) were deemed of sufficiently high quality and free of detrimental artifacts. The images were segmented using the in-house developed and released MOOSE software version 3.0.13^25^, which was trained on a cohort of 1683 individual cases^39^, not including the presented cohort. For each subject and dataset (Test, Retest), MOOSE was used to automatically delineate 135 organs from the CT images. Since the CT and PET images were reconstructed with different voxel and matrix sizes, any CT-based multi-organ segmentation mask must be resampled – using a nearest-neighbor interpolation – to match the PET voxel and matrix sizes before extracting complementary regional PET and CT image quantification. Of note, prior to resampling, no additional PET-CT registration was performed for the combined PET/CT data.

In total, 11 biometric parameters were retrieved from both the PET and CT images per organ and subject. These parameters included: six parameters from the CT image (mean, standard deviation (STD), median, minimum, maximum tissue densities in HU, and organ volume), and five corresponding regional values from the matching PET images (mean, STD, median, minimum, and maximum SUV). We evaluated the mean and STD across the 48 subjects at Test and Retest (Fig. 1) as estimates of the normative, cohort-specific organ parameters at both time points. All data processing and metric extraction were done with Python 3.10, utilizing the SimpleITK^40,41^ package.

While the STD of the normative values across the 135 segmented regions can be estimated from each individual organ (Table S2), we limit our robustness analysis – that is, the Test/Retest variability – to 10 target organs that are known to be involved in a number of prevalent systemic diseases: liver, spleen, lungs (all five lobes), brain, pancreas, kidneys (left and right), heart, subcutaneous fat, visceral fat, and skeletal muscle. Volumes of the latter three tissues were defined at the vertebral L3 level. Here, the L3 level was selected as a well-established surrogate location for global body-composition measurements^42–44^ and more efficient delineation and verification of the fat and muscle segments for subsequent model training.

To evaluate potential sex-based differences in imaging-derived readouts, we performed a group-level comparison using the unpaired Student’s t-test, as a statistical assessment of between-group variability. Further, we assessed the repeatability of the Test and Retest scans at both the group and the subject levels. For the group analysis, we calculated the region-based averages across all 48 subjects, from which we derived statistical differences between Test and Retest readouts using the relative %-difference and the coefficient of variation (CV). We then used a paired Student’s t-test to assess the statistical significance of within-subject repeatability since each subject contributes a directly linked Test-Retest pair. Lastly, the Intraclass Correlation Coefficient (ICC) was used to evaluate repeatability for each organ and parameter^45,46^, specifically ICC(3,1), which assesses absolute agreement in repeated measures from the same subjects under identical conditions^46^. For the subject-level analysis, we computed the relative %-difference between the Test and Retest scans for each region on a per-subject basis, averaging these differences across the 48 subjects.

In principle, normative data (Table S2), as included in this healthy cohort, can be used to assess aberrations of tissue metabolism, organ volume, or organ density, as available from cohort-specific readouts of PET (SUV) and CT ([mL], [HU]). Disease-related aberrations can be detected as long as their quantitative impact on CT or PET readouts is larger than the Test/Retest variability. Here, we computed z-scores to highlight subject-specific deviations from the established norm, such as SUV, HU, and volume for each of the 135 segmented organs. The following formula (**I**) was applied to each organ parameter set to compute the z-score, denoted as $z$:

I $$z=\frac{{mea}{n}_{{subject}}-{mea}{n}_{{cohort}}}{{ST}{D}_{{cohort}}}$$

The resulting z-scores can be visualized on an organ and voxel level^47–49^. For illustration purposes, we generate an organ atlas that highlights z-scores as deviations from the norm with 2 STDs as a cut-off to filter out noise.

To further expand the usability of this dataset in terms of chronobiological assessments, we report the start time of the Test and Retest PET/CT acquisition for each subject in Table 1. The time of day column indicates whether scans were performed before noon, in the afternoon, or the evening.

**Table 1:** Start times of examinations of Test and Retest as a reference for future analyses of the chronobiological effects on PET/CT scans for each subject.

Subject	Test		Retest	
	Time (Start)	Time of day	Time (Start)	Time of day
001	15:58:28	afternoon	12:46:17	afternoon
002	17:17:01	afternoon	17:21:05	afternoon
003	13:55:02	afternoon	13:09:10	afternoon
004	16:37:10	afternoon	12:56:56	afternoon
005	14:17:29	afternoon	16:04:23	afternoon
006	16:01:43	afternoon	15:53:00	afternoon
007	15:10:48	afternoon	14:28:24	afternoon
008	14:04:32	afternoon	12:45:40	afternoon
009	18:24:37	evening	18:14:18	evening
010	13:14:06	afternoon	09:34:38	before noon
011	15:52:35	afternoon	17:38:24	afternoon
012	15:59:17	afternoon	13:55:08	afternoon
013	15:57:18	afternoon	15:37:58	afternoon
014	13:23:12	afternoon	15:53:12	afternoon
015	17:08:27	afternoon	12:23:46	afternoon
016	17:29:06	afternoon	10:04:17	before noon
017	11:04:53	before noon	09:32:23	before noon
018	13:49:17	afternoon	12:43:36	afternoon
019	16:40:03	afternoon	13:22:52	afternoon
020	17:09:52	afternoon	13:46:48	afternoon
021	15:41:28	afternoon	19:57:30	evening
022	15:39:12	afternoon	14:57:31	afternoon
023	15:15:59	afternoon	11:04:37	before noon
024	14:55:27	afternoon	10:18:26	before noon
025	17:34:44	afternoon	16:46:47	afternoon
026	16:50:27	afternoon	09:41:07	before noon
027	14:25:57	afternoon	17:11:08	afternoon
028	15:37:43	afternoon	09:55:02	before noon
029	09:30:54	before noon	10:39:49	before noon
030	15:25:34	afternoon	20:19:10	evening
031	10:01:38	before noon	19:55:45	evening
032	15:19:21	afternoon	16:17:25	afternoon
033	14:30:51	afternoon	15:45:34	afternoon
034	17:15:49	afternoon	16:02:05	afternoon
035	12:46:19	afternoon	09:35:58	before noon
036	13:16:54	afternoon	22:20:33	evening
037	14:24:05	afternoon	20:10:37	evening
038	10:34:50	before noon	20:01:25	evening
039	13:45:07	afternoon	12:24:07	afternoon
040	15:12:22	afternoon	19:40:49	evening
041	17:35:51	afternoon	20:13:09	evening
042	16:53:33	afternoon	20:42:36	evening
043	10:16:23	before noon	21:52:32	evening
044	17:40:50	afternoon	19:36:40	evening
045	10:02:05	before noon	19:54:58	evening
046	13:55:28	afternoon	21:05:13	evening
047	11:25:09	before noon	21:21:37	evening
048	19:01:11	evening	19:23:33	evening

The time of day is categorized as: before noon: <12:00:00, afternoon: >12:00:00, evening: >18:00:00.

Facial anonymization of the image data was performed prior to data sharing. Defacing was applied to the PET and CT images of each subject by first extracting a facial segmentation mask from the CT using the “face” task of TotalSegmentator version 2.10.0^50^. This mask was then applied to both the CT and PET images; for PET, the mask was coregistered using nearest-neighbor interpolation. In both modalities, the masked facial region was downsampled and subsequently upsampled to obscure identifiable features. The pixelated region was reinserted into the corresponding original image using the segmentation mask, effectively replacing the original facial anatomy with an unrecognizable version. All image analysis and quantification presented in this study were performed on the original, non-defaced PET and CT images.

## Data Records

All imaging data are stored in anonymized and defaced NIfTI format. The entire image dataset includes the CT images, the static PET images at 57–62 min post-injection, and the segmentations generated by MOOSE with a total size of approximately 20.2 GB. It can be accessed unrestricted through Zenodo under the Creative Commons Attribution 4.0 International (CC BY 4.0) license, following this link: https://zenodo.org/records/16364694 or by searching for the term “QUADRA_HC” on Zenodo^51^.

**Fig. 3:**
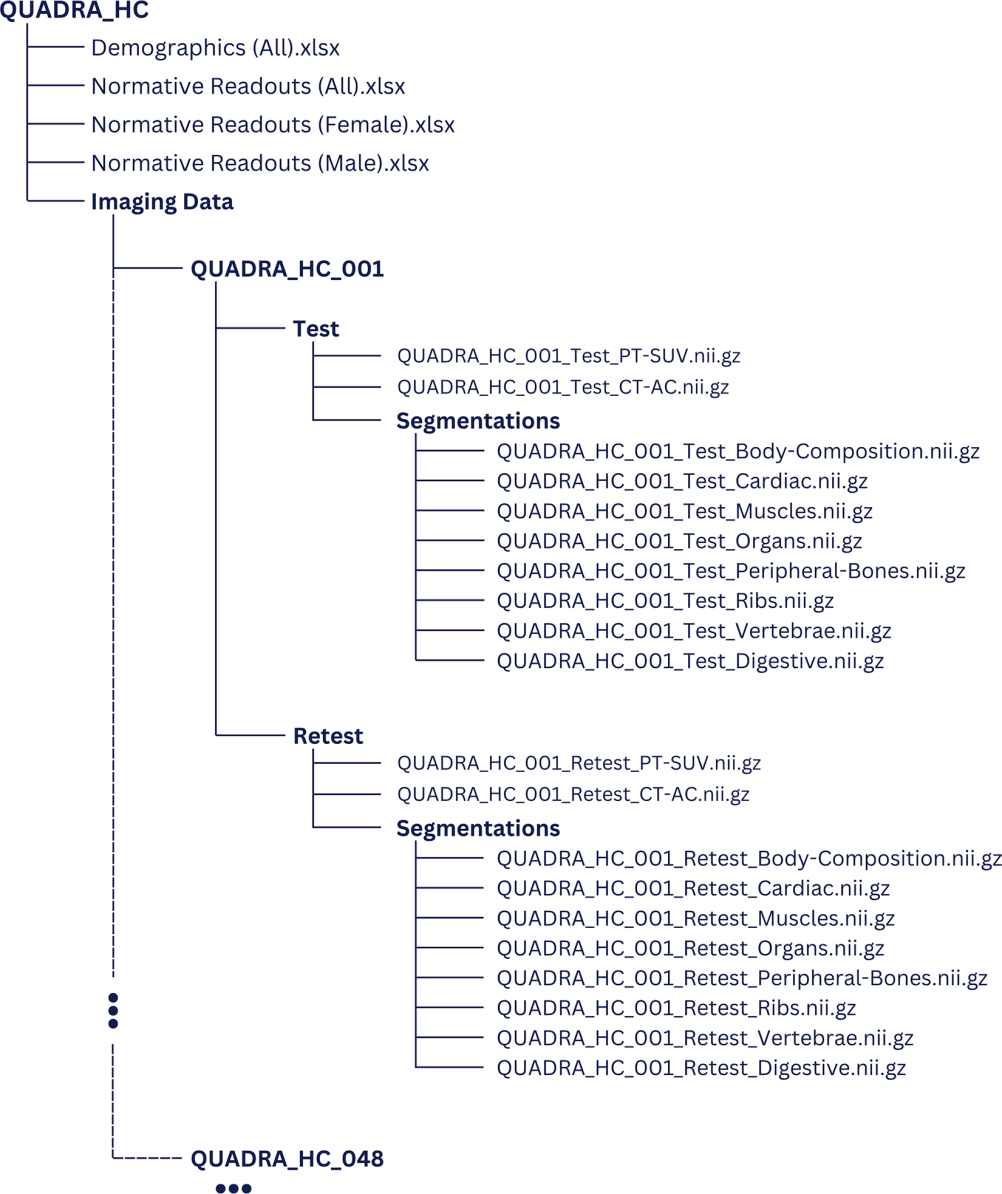
Folder structure of the QUADRA_HC dataset. Each subject folder, identified by the subject ascending ID, QUADRA_HC_001 to QUADRA_HC_048, contains a Test and Retest folder in which the PET image (scaled to SUV), the low-dose CT used for attenuation correction, and a folder containing the segmentations are placed. The segmentation files contain 135 distinct regions of interest as provided by MOOSE^25^. All image and segmentation files are provided in anonymized and defaced NIfTI format. The demographics and normative readouts of the cohort for all, all female, and all male subjects are provided at the top level of the QUADRA_HC folder.

The demographics for this cohort are summarized in Table S1 of this manuscript. Organ-based readouts for the entire cohort are presented in Table S2, Table S3, and Table S4. The contents of these tables are also provided in the repository^51^ as Excel files along with the image data, for download as **Demographics (All).xlsx,** **Normative Readouts (All).xlsx,** **Normative Readouts (Male).xlsx**, and **Normative Readouts (Female).xlsx**, respectively (Fig. 3).

**Fig. 4:**
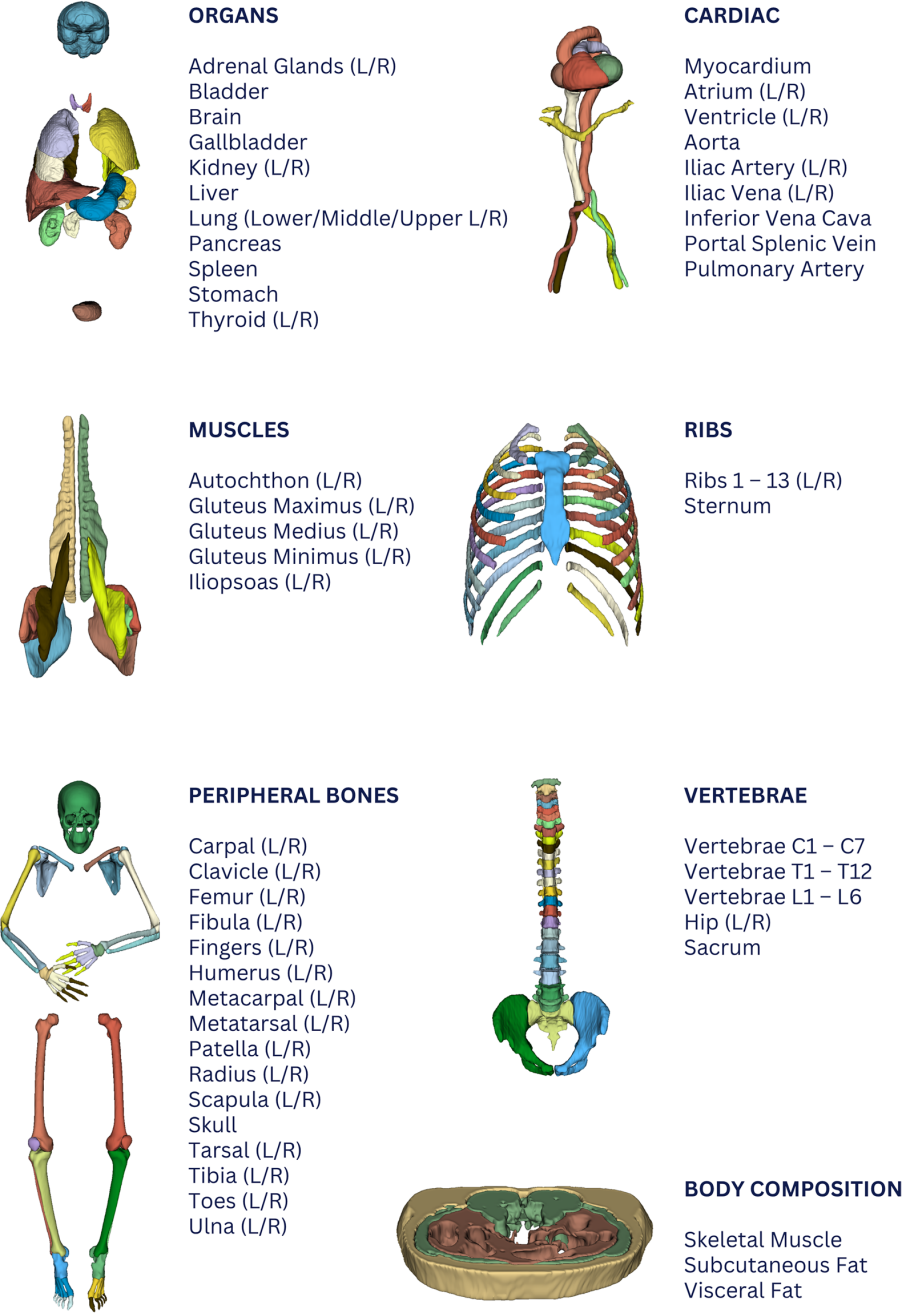
Overview of all organ/tissue segmentations as extracted by MOOSE^25^. For the purpose of this report, we share mean, STD, median, minimum, and maximum readouts for CT and PET, as well as the organ/tissue volume for each subject at Test and Retest (see main text for details).

Figure 3 shows the directory structure of the imaging and data of this study protocol. The imaging-data folder contains one folder per subject, split into “Test” and “Retest” acquisitions, each containing the corresponding CT and PET images along with anatomical segmentations in anonymized and defaced NIfTI format. The anatomical segmentations are grouped within eight different multi-label files corresponding to distinct segmentation classes (Fig. 4): “Body-Composition,” “Cardiac,” “Muscles,” “Organs,” “Peripheral Bones,” “Ribs,” “Digestive,” and “Vertebrae”.

## Technical Validation

**Fig. 5:**
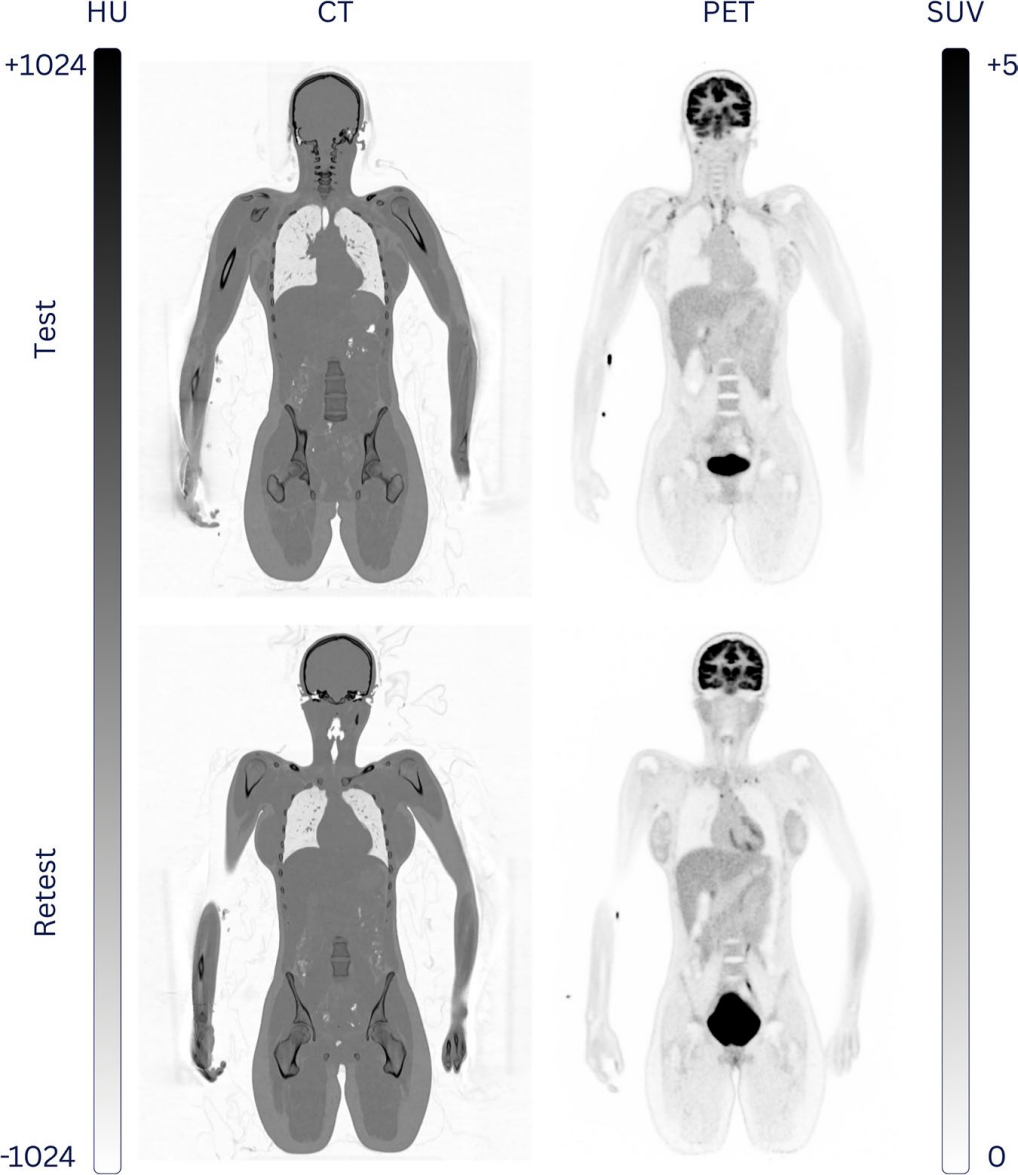
Example of WB-FDG-PET/CT image quality for subject QUADRA_HC_001 (20-y/o female) for Test and Retest that were 35 days apart.

**Fig. 6:**
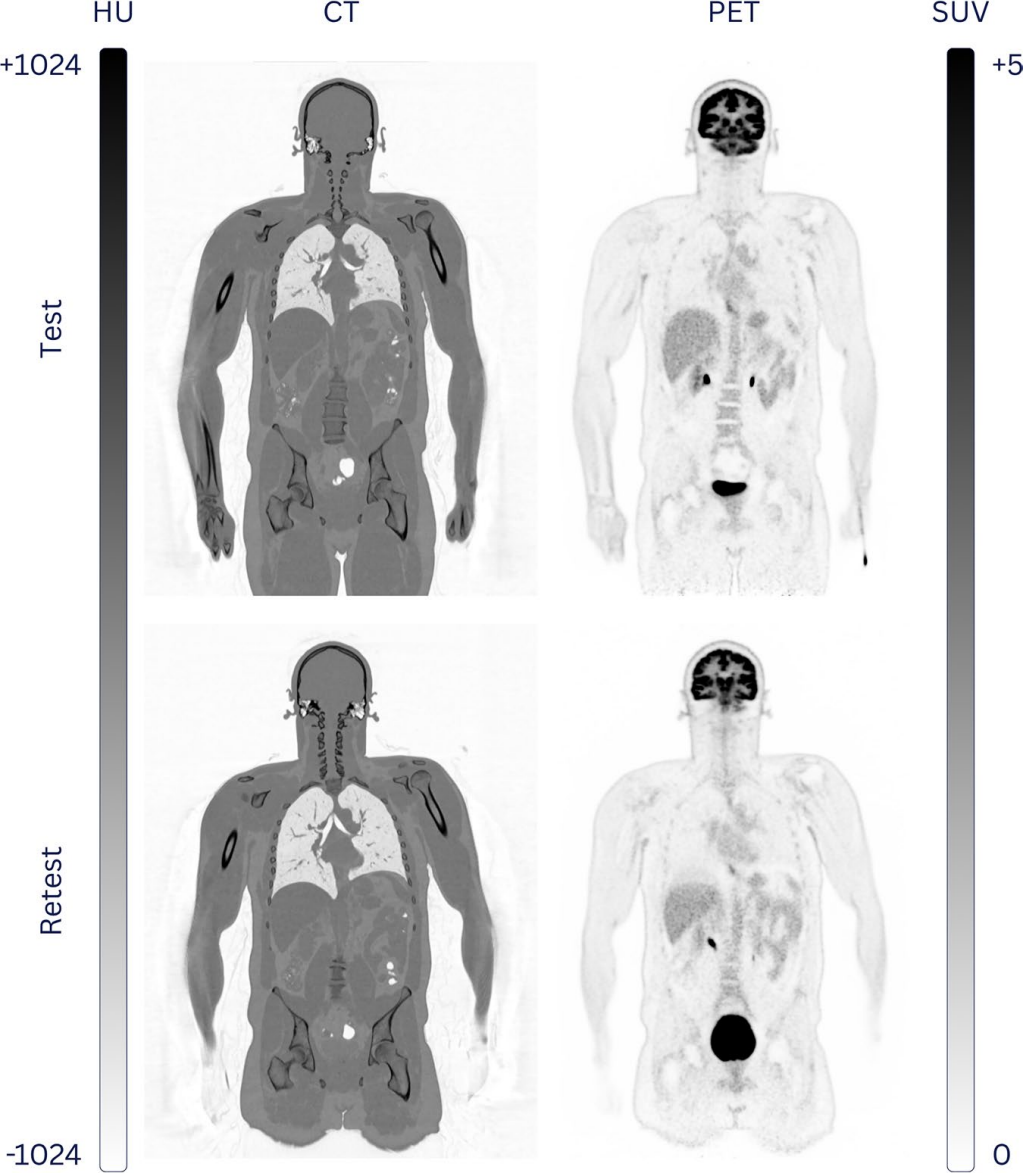
Example of WB-FDG-PET/CT image quality for subject QUADRA_HC_002 (40-y/o male) for Test and Retest that were 35 days apart.

Our technical validation was limited to 48 healthy controls without subsequent incidental findings. The inclusion of the remaining 48 healthy controls and completion of the study protocol for each participant (Fig. 1) took 312 days. All subjects underwent WB-FDG-PET/CT imaging twice, with a mean time difference of (38 ± 10) days. Examples of the CT and PET image quality of a female and male healthy subject undergoing Test and Retest FDG-PET/CT imaging are shown in Figs. 5, 6, respectively.

### Normative organ readouts

A detailed list of all 135 regions that were segmented by our in-house segmentation tool, MOOSE^25^ is shown in Fig. 4. Table S2 lists the normative values extracted from the Test and Retest PET/CT examination that were averaged across the 48 included healthy controls. These values correspond to values reported in previous studies of SUV in healthy subjects^52–54^. However, the reported literature values stem mostly from a restricted number of volumes of interest only. Furthermore, some of their reported values were extracted from specific regions within target organs rather than from the entire organ. In contrast to these reports, our readouts – though confined to Caucasian controls - relate to a significantly larger number of target organs and tissues, and they provide whole-organ context and analyses.

**Fig. 7:**
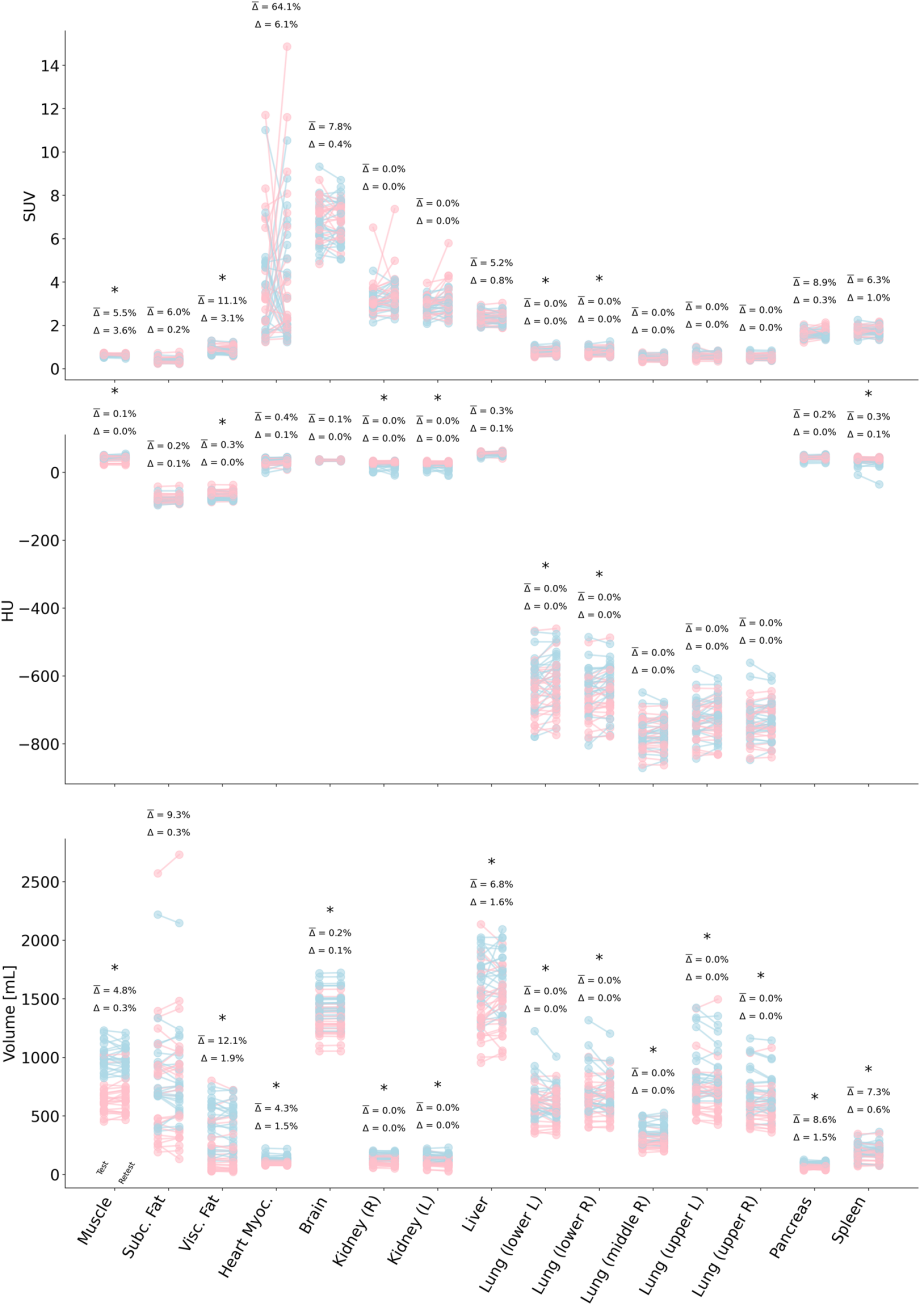
Slope plots showing Test (left side of each slope) and Retest (right side) measurements for PET activity [SUV], CT density [HU], and CT volume [mL] across 10 reference regions in 48 healthy subjects (R = right, L = left). Male subjects are shown in light blue, females in light pink. Above each plot, the mean intra-subject percentage difference (Δ̄%) is indicated; below, the group-level mean absolute percentage difference (Δ%). The asterisks indicate statistically significant differences between males and females. Overall, organ-specific mean values remained stable between Test and Retest, with only minor variability, supporting the robustness of these metrics in healthy individuals.

While normative values were computed across all segmented organs and tissues, the assessment of potential sex differences focuses specifically on the aforementioned subset of organs selected for repeatability analysis: liver, spleen, lungs (including all five lobes), brain, pancreas, kidneys (left and right), heart, subcutaneous fat, visceral fat, and skeletal muscle. The unpaired t-test analysis revealed statistically significant differences between male and female subjects across all readout parameters in several organs, as denoted by the asterisk in Fig. 7. The mean SUV was significantly different between male and female cohorts in the skeletal muscle (p < 0.01), myocardium (p < 0.01), right lower lung lobe (p = 0.02), and left lower lung lobe (p = 0.02), whereas good agreement was observed in all other regions. Differences were more pronounced for Hounsfield units and volumes, where most regions showed significant disparities between the male and female cohorts. Table S3 and Table S4 report the normative readout values for the male and female gender matched subcohorts, respectively, in addition to the complete coverage of all 48 subjects in Table S2.

### Repeatability assessment

In order to employ normative values as a reference to compare potential disease-induced metabolic or tissue density aberrations, robustness values must be established. In other words, any aberration caused by or correlated with a systemic disease must cause deviations from a pre-established norm that are significantly different from the STD of the normative data. Therefore, we have performed test and retest scans as Test and Retest. Figure 7 visualizes the organ readouts at Test and Retest with slope plots of selected target organs from the healthy cohort (N = 48). The plots demonstrate a strong agreement between timepoints for individual subjects and their CT-based density and volume as well as PET-based SUV readouts, showing mostly shallow and tightly packed slopes indicating only minor changes between Test and Retest, not only at the subject level, but also at the group level. Exceptions were observed in the renal and myocardial SUV. These visually assessed results underscore the robustness of the measured parameters and support their application as stable normative baselines in clinical and research contexts, which was confirmed by our statistical analysis outlined below.

Overall, the SUV exhibited a strong consistency, both at the group and the subject levels (Table 2), thereby reaffirming the robustness of PET-based quantitative assessments. In terms of group-level findings, the SUV of the key organs varied by less than 5%, except for the kidneys (5.3% <  = Δ% <  = 6.5%) and the heart (Δ% = 6.1%), both of which exert an intrinsically high variability of FDG uptake. The calculated ICC(3,1) values supported these observations, with most organs showing excellent agreement (ICC > 0.95), except for skeletal muscle (ICC = 0.71), heart (ICC = 0.01), kidneys (ICC = 0.49 left, 0.39 right) and pancreas (ICC = 0.43), indicating moderate and poor repeatability, potentially due to the motion-induced spatial intra-scan mismatch between PET and CT. Furthermore, the SUV of skeletal muscle was significantly different between scans (p < 0.05), likely due to a difference in muscle activity shortly before the scans. Subject-level analysis corroborated these observations, indicating that the majority of organ-based SUV readouts remained stable across repeated scans (Δ% < 9%). We did observe a noticeable change in Δ% of 11% (subject level) in the SUV readout of visceral fat, which was likely due to changes in the weight of the subjects between the Test and Retest scans. The calculated CV from the Test (11.4%) and Retest (10.9%) for PET images in the liver (not reported in the tables) was well below the reported threshold of 15%, as recommended in the EARL guidelines^55,56^.

**Table 2:** Test/Retest variability of mean SUV of the selected key organs of interest.

Organ SUV	Test [SUV]	Retest [SUV]	Group					Subject
			Δ%	t-test (paired)		ICC		
				t-value	p-value	(3,1)	CI95%	Δ%
Skeletal Muscle	0.6 ± 0.1	0.6 ± 0.1	3.6	3.63	0	0.71	0.54–0.83	5.5
Subcutaneous Fat	0.4 ± 0.1	0.4 ± 0.1	0.2	0.13	0.89	0.96	0.93–0.98	6
Visceral Fat	0.9 ± 0.2	0.8 ± 0.2	3.1	1.65	0.1	0.78	0.63–0.87	11.1
Heart Myocardium	3.8 ± 2.4	4 ± 3.1	6.1	−0.41	0.68	0.01	−0.27–0.29	64.1
Brain	6.8 ± 1.0	6.8 ± 0.9	0.4	0.27	0.79	0.71	0.54–0.83	7.8
Kidney (left)	2.9 ± 0.4	3.1 ± 0.6	6.5	−2.52	0.02	0.49	0.24–0.68	11.9
Kidney (right)	3.2 ± 0.7	3.4 ± 0.8	5.3	−1.48	0.14	0.39	0.12–0.6	13.1
Liver	2.3 ± 0.3	2.3 ± 0.3	0.8	0.83	0.41	0.82	0.7–0.89	5.2
Lung (lower left)	0.8 ± 0.2	0.8 ± 0.2	2	−1.44	0.16	0.88	0.8–0.93	7.1
Lung (lower right)	0.8 ± 0.2	0.8 ± 0.2	0.7	−0.47	0.64	0.88	0.79–0.93	7.1
Lung (middle right)	0.5 ± 0.1	0.5 ± 0.1	0.8	−0.63	0.53	0.92	0.85–0.95	6.3
Lung (upper left)	0.6 ± 0.1	0.6 ± 0.1	0.8	0.39	0.7	0.78	0.65–0.87	8.5
Lung (upper right)	0.5 ± 0.1	0.5 ± 0.1	0.8	−0.83	0.41	0.95	0.91–0.97	4.8
Pancreas	1.6 ± 0.2	1.6 ± 0.2	0.3	−0.15	0.88	0.43	0.17–0.63	8.9
Spleen	1.8 ± 0.2	1.7 ± 0.2	1	0.83	0.41	0.72	0.55–0.83	6.3

Δ% (group level) reflects the relative difference between the mean Test and Retest intensities across all subjects. Δ% (subject level) represents the average of individual Test-Retest percentage differences computed per subject. Repeatability was considered good for: Δ% < 6.6% on the group level, Δ% < 41% on the subject level (thresholds were determined by: mean Δ% + 2 SD), and statistical significance of p > 0.05. Values not meeting these criteria are highlighted in bold text. Group-level analysis showed high consistency of SUV measurements (Δ% < 5%) for most organs, with the exception of the kidneys (Δ% = 5.3–6.5%) and heart (Δ% = 6.1%), which are known to exhibit high physiological variability. ICC(3,1) values confirmed excellent repeatability (ICC > 0.95) in most regions, but were notably lower in skeletal muscle (ICC = 0.71), pancreas (ICC = 0.43), and especially the heart (ICC = 0.01) and kidneys (ICC = 0.49 left, 0.39 right). Subject-level repeatability was similarly strong (Δ% < 9%) for most organs, except visceral fat (Δ% = 11.1%), likely reflecting weight-related changes.

For tissue density – measured in HU, the group-level comparisons (Table 3) showed consistency across all target regions with small deviations of Δ% <2%. Of note, the %-differences were highest for the lower lung lobes at ~3%. The paired t-tests revealed significant differences in the lungs, heart, and subcutaneous fat regions. ICC(3,1) values indicated excellent repeatability in most tissues (ICC > 0.95), with slightly lower values for the heart (ICC = 0.85) and brain (ICC = 0.79), suggesting moderate consistency in these regions. These differences were likely driven by breathing (lung area) and motion (heart) artefacts in either examination, and presumably by weight changes and alterations in body composition between the two PET/CT scans. However, the correlation between tissue density and weight changes needs to be further investigated. On the subject level, the lung lobes expressed the highest percentage differences in tissue density of 4.4%–7.9%, with the remaining organs staying well below 1%. This observation confirmed the motion-induced deviations again, as the lower lung lobes showed differences on the higher end of the determined range.

**Table 3:** Test/Retest variability of mean CT-based density (HU) of the selected key organs of interest.

Organ HU	Test [HU]	Retest [HU]	Group					Subject
			Δ%	t-test (paired)		ICC		
				t-value	p-value	(3,1)	CI95%	Δ%
Skeletal Muscle	42 ± 7	42 ± 7	0.0	−1.43	0.16	0.96	0.93–0.98	0.1
Subcutaneous Fat	−80 ± 10	−80 ± 10	0.1	−2.06	0.05	0.97	0.96–0.99	0.2
Visceral Fat	−68 ± 11	−69 ± 11	0.0	0.64	0.53	0.95	0.91–0.97	0.3
Heart Myocardium	32 ± 9	33 ± 9	0.1	−1.97	0.05	0.85	0.75–0.91	0.4
Brain	35 ± 2	35 ± 1	0.0	0.77	0.44	0.79	0.65–0.87	0.1
Kidney (left)	25 ± 7	23 ± 10	0.2	1.6	0.12	0.63	0.43–0.78	0.4
Kidney (right)	25 ± 8	25 ± 9	0.1	0.5	0.62	0.64	0.44–0.78	0.4
Liver	54 ± 4	55 ± 4	0.1	−1.15	0.25	0.60	0.38–0.75	0.3
Lung (lower left)	−634 ± 73	−621 ± 74	3.3	−2.41	0.02	0.87	0.78–0.92	7.9
Lung (lower right)	−654 ± 67	−644 ± 67	2.7	−2.37	0.02	0.91	0.84–0.95	6.3
Lung (middle right)	−772 ± 44	−769 ± 43	1.3	−1.62	0.11	0.95	0.92–0.97	4.4
Lung (upper left)	−731 ± 53	−725 ± 54	1.8	−1.82	0.08	0.93	0.87–0.96	5.9
Lung (upper right)	−737 ± 54	−731 ± 53	1.9	−2.01	0.05	0.94	0.9–0.97	5.2
Pancreas	43 ± 5	44 ± 5	0.0	−0.72	0.47	0.82	0.7–0.9	0.2
Spleen	37 ± 9	35 ± 12	0.1	1.67	0.1	0.86	0.76–0.92	0.3

Δ% (group level) reflects the relative difference between the mean Test and Retest intensities across all subjects. Δ% (subject level) represents the average of individual Test-Retest percentage differences computed per subject. Repeatability was considered good for: Δ% < 3% on the group level, Δ% < 8% on the subject level (thresholds were determined by: mean Δ% + 2 SD), and statistical significance of p > 0.05. Values not meeting these criteria are highlighted in bold text. Group-level Δ% was < 2% for most organs, with slightly higher variability observed in the lower lung lobes (~3%). ICC(3,1) values were > 0.95 in most tissues, though moderately lower in the heart (ICC = 0.85) and brain (ICC = 0.79), likely due to motion artefacts and physiological noise. Subject-level differences were generally minimal, with the exception of the lungs, where Δ% ranged from 4.4% to 7.9%, again reflecting respiratory motion variability.

Both CT (HU) and PET (SUV) exhibit generally low group‐level variability (<6% for most organs), indicating good overall repeatability, supported by ICC measures. At the single‐subject level, they remain stable in most tissues. Still, each modality has its expected outliers: PET shows a significant day‐to‐day fluctuation in the heart (Δ% ≈ 64%) and generally lower ICC values, most likely due to the metabolic variability of subjects. In comparison, tissue density exhibits relatively high variability in the lungs (Δ% up to 7.9%). Apart from these regions, both imaging modalities exhibit good repeatability.

Organ volume measurements proved to be the most repeatable, with group-level analyses (Table 4) indicating less than 3.5% variation between Test and Retest scans for almost all target organs. Small but significant differences appeared in the brain (p = 0.04) and kidney (p = 0.04) in the analyses. Repeatability was further confirmed by high ICC(3,1) values across all organs (ICC > 0.95, except in the liver and lung lobes), including the brain and kidneys, which showed significant group-level differences but retained strong agreement in volumetric measurements. In the subject-level assessment, the percentage differences in body-composition volumes and the lungs were more pronounced, as weight changes and breathing artefacts had a greater impact at the subject level.

**Table 4:** Test/Retest variability measurements of CT-based organ volume of the selected key organs of interest.

Organ Volume	Test [mL]	Retest [mL]	Group					Subject
			Δ%	t-test (paired)		ICC		
				t-value	p-value	(3,1)	CI95%	Δ%
Skeletal Muscle	816 ± 217	818 ± 207	0.3	−0.31	0.76	0.97	0.95–0.98	4.8
Subcutaneous Fat	795 ± 455	797 ± 467	0.3	−0.2	0.84	0.99	0.98–0.99	9.3
Visceral Fat	315 ± 228	309 ± 223	1.9	0.88	0.38	0.98	0.96–0.99	12.1
Heart Myocardium	116 ± 28	118 ± 28	1.5	−1.54	0.13	0.96	0.93–0.98	4.3
Brain	1377 ± 147	1378 ± 148	0.1	−2.09	0.04	1.00	1.0–1.0	0.2
Kidney (left)	129 ± 44	125 ± 42	3.5	2.17	0.04	0.95	0.91–0.97	8.7
Kidney (right)	132 ± 39	129 ± 39	1.8	1.46	0.15	0.96	0.93–0.98	6.6
Liver	1534 ± 283	1559 ± 267	1.6	−1.19	0.24	0.87	0.78–0.93	6.8
Lung (lower left)	613 ± 153	594 ± 131	3.2	1.55	0.13	0.83	0.71–0.9	10.4
Lung (lower right)	683 ± 176	669 ± 157	2.1	1.22	0.23	0.89	0.81–0.94	8.5
Lung (middle right)	323 ± 80	323 ± 80	0.1	0.08	0.93	0.97	0.94–0.98	4.9
Lung (upper left)	783 ± 235	770 ± 226	1.6	1.41	0.17	0.96	0.94–0.98	6.2
Lung (upper right)	636 ± 179	627 ± 173	1.4	1.4	0.17	0.97	0.95–0.98	5.4
Pancreas	79 ± 22	81 ± 22	1.5	−1.05	0.3	0.94	0.89–0.96	8.6
Spleen	186 ± 63	185 ± 65	0.6	0.39	0.7	0.95	0.91–0.97	7.3

Δ% (group level) reflects the relative difference between the mean Test and Retest intensities across all subjects. Δ% (subject level) represents the average of individual Test-Retest percentage differences computed per subject. Repeatability was considered good for: Δ% < 3.5% on the group level, Δ% < 13% on the subject level (thresholds were determined by: mean Δ% + 2 SD), and statistical significance of p > 0.05. Values not meeting these criteria are highlighted in bold text. The group-level Δ% was < 3.5% for nearly all organs. Statistically significant group-level differences were observed in the brain and kidneys (both p = 0.04), but these did not compromise overall repeatability, as ICC(3,1) values remained > 0.95 in most regions. Slightly lower ICCs were seen in the liver and lung lobes, and subject-level differences were more pronounced in organs sensitive to weight fluctuations and respiration, such as visceral fat (Δ% = 12.1%) and lungs.

Despite these described slight variations, the study exhibits excellent overall repeatability, confirming that Test and Retest scans provide reliable and consistent quantitative measurements across all three parameters. Exceptions, such as myocardial and renal SUV fluctuations, were to be expected, such as slight but noticeable changes in volume due to potential weight changes of subjects.

### Application of normative readouts and findings

**Fig. 8:**
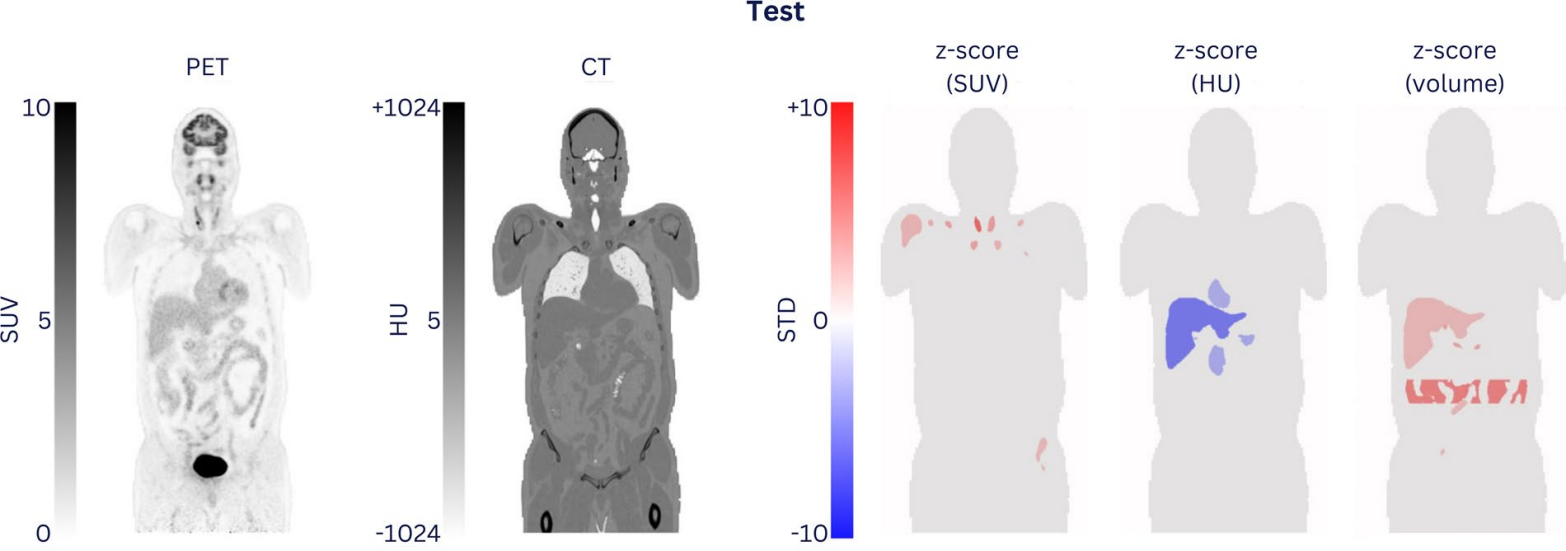
Example of organ-based aberrations of the norm: 55-y/o male (QUADRA_HC_049; excluded from the shared dataset) was found with thyroid cancer. The organ-based readouts of the subject were compared to the respective normative values derived from the cohort of 48 healthy controls (Table S2). From left to right: coronal CT, coronal PET, organ-based aberrations (z-score) for SUV, density (HU), and volume. As expected, the SUV of the thyroid changed by more than 2 STD. In addition, an increased liver volume and lower liver tissue density were observed when comparing the patient to the average of the corresponding cohort. Of note, this figure reports organ-based deviations of the norm (>2STD) for illustration purposes only; potentially disease-induced changes cannot be confirmed with the data at hand.

The example of a patient-specific z-score atlas is shown in Fig. 8. These data articulate the value of a more systemic analysis of WB-PET/CT readouts in the presence of malignant disease. While it relays corollaries to the presence of a systemic disease, the potential application of such patient maps should be reviewed in more detail as the availability of normative databases expands.

Finally, of all subjects enrolled, two subjects presented with incidental findings on their 1st (Test) WB-FDG-PET/CT scan: a T2N0M0 papillary thyroid carcinoma and an adenocarcinoma of the lung stage T1cN0M0. Both subjects were successfully treated for their cancers, and their data were removed from the shared data set. These incidental findings also underscore the potential of systematic screening in uncovering clinically relevant conditions that might otherwise remain undiagnosed. Interestingly, the 4% incidence in here is in the range of incidences reported by earlier screening studies of asymptomatic subjects undergoing static WB-FDG-PET/CT imaging in Japan^57,58^.

## Usage Notes

All imaging data are presented in NIfTI format, ensuring the privacy of the study participants while allowing for easy use in further analysis. This format can be opened with most visualization software, including 3D Slicer (https://www.slicer.org/) and ITK-SNAP (http://www.itksnap.org/pmwiki/pmwiki.php). DICOM to NIfTI conversion was performed using the software dcm2niix^59^, and all image processing was conducted using Python and the SimpleITK^40,41^ package.

More advanced stratification and analysis beyond sex-matched subcohorts can be achieved by referencing the subject information in the demographics in Table S1.

### Limitations

This dataset is made of whole-body [^18^F]FDG-PET/CT imaging data acquired with a Siemens Quadra PET/CT system of 48 healthy controls. It includes paired test-retest scans and organ-level segmentations. The cohort contains equal male and female subjects across a reasonably limited range of ages and BMIs; however, the sample size is still low (n = 48), thus, limiting the generalizability of any subgroup analyses (e.g., stratification by age, BMI, sex). Further, all organ segmentations were generated automatically using the MOOSE algorithm^25^. Due to its previously reported high accuracy, we did not perform additional visual verification of each of the 135 segmentations per subject. Given the extended imaging protocol, involuntary intra-scan motion of subjects may cause local misalignments of PET and CT information that may translate into a bias of the attenuation-corrected PET data (despite the lack of visible artifacts) as well as slight misalignments of the CT-based segmentation mask and corresponding tracer uptake on PET. One specific area of recurring mismatch we observed was the hands, likely attributed to the involuntary movement during the long acquisition time. However, these misalignments appeared to have only localized effects and did not substantially affect image quality. Bespoke misalignment of segmentation masks is potentially more relevant when analysing PET data derived from the last 5 minutes of the emission scan, which are acquired 57 minutes after the initial CT scan. Here, motion-induced misalignments are more critical in central regions such as the torso, mainly observed in the lungs due to breathing. In contrast, mismatches in the extremities are less likely to impact quantitative analyses.

Finally, the release of raw listmode PET data was not feasible for this study. Each scan generated ~180 GB of listmode data, amounting to >15 TB across the entire cohort, which exceeds the capacity of commonly used public repositories (e.g., Zenodo limits are 200 GB). Moreover, residual metadata embedded in listmode headers (e.g., acquisition times, system identifiers, protocol strings) require vendor-specific anonymisation and institutional review to ensure compliance with data governance regulations.

Within these limitations, these data support the development of a normative reference for CT-based body composition and systemic FDG uptake in healthy controls and thus offer valuable insights into the repeatability in multi-organ whole-body FDG-PET/CT analysis. By making this dataset openly available, we aim to facilitate future research into systemic imaging biomarkers and support methodological developments in quantitative PET/CT analysis of health and disease.

## Supplementary information

Supplementary Information

## Data Availability

The dataset is available on Zenodo at https://zenodo.org/records/16364694 or 10.5281/zenodo.16364694, and can also be found by searching for “QUADRA_HC”^51^.

## Code availability

The MOOSE code is open-source and available online through GitHub at https://github.com/ENHANCE-PET/MOOSE, along with extensive documentation. The image dataset is available for download at Zenodo via unrestricted access under the Creative Commons Attribution 4.0 International (CC BY 4.0) license, following this link: https://zenodo.org/records/16364694 or by searching for the term “QUADRA_HC” on Zenodo^51^. No separate repository is provided for storing the code used for analysis. All analysis was performed with Python using the SimpleITK package and the available functions to extract organ readouts and perform computational operations. 3D Slicer and ITK-SNAP can be downloaded free of charge following the previously mentioned links.
