# Supplementary material for: Whole-Body [18F]FDG-PET/CT Imaging of Healthy Controls: Test/Retest Data for Systemic, Multi-Organ Analysis
Source: Sci Data. 2025 Oct 29;12:1707. doi: 10.1038/s41597-025-05997-4 (PMC12572201; doi:10.1038/s41597-025-05997-4)
Supplement: Supplementary file 1 — Supplementary Information [file 41597_2025_5997_MOESM1_ESM.docx]

# Supplementary Tables

**Table S1.** Demographics of the 48 healthy controls participating in the test/retest FDG-PET/CT imaging protocol. Key information for the Test and Retest PET/CT imaging protocols for all 48 subjects include: subject weight [kg], injected FDG activity [MBq], post injection time (t_PI_), time differences [min] between “Initiation” and “Test” scan (Pre-Scan), “Test” and “Retest” (Scan) as well as “Retest” and “Release” (Post-Scan) in days. Changes in subject weight between “Test” and “Retest” scans are reported as relative %-changes. F – female, M – male, y-years, BMI – body mass index.

| **Subject** | **Sex** | **Height  [m]** | **Age  [y]** | **Test** | | | | **Retest** | | | | **Difference** | | | |
| --- | --- | --- | --- | --- | --- | --- | --- | --- | --- | --- | --- | --- | --- | --- | --- |
|  |  |  |  | **Weight  [kg]** | **BMI  [kg/m²]** | **t_PI_  [min]** | **Inj. A.  [MBq]** | **Weight  [kg]** | **BMI  [kg/m²]** | **t_PI_  [min]** | **Inj. A.  [MBq]** | **Weight  %** | **Pre-Scan  [d]** | **Scan  [d]** | **Post-Scan  [d]** |
| 001 | F | 1.7 | 20 | 56 | 21 | 57 | 106 | 53 | 20 | 61 | 113 | 5.3 | 66 | 35 | 111 |
| 002 | M | 1.8 | 40 | 84 | 27 | 56 | 99 | 86 | 27 | 57 | 96 | 2.0 | 73 | 35 | 85 |
| 003 | M | 1.9 | 41 | 89 | 26 | 54 | 103 | 87 | 24 | 57 | 117 | 2.5 | 35 | 98 | 90 |
| 004 | F | 1.9 | 43 | 89 | 25 | 57 | 107 | 89 | 25 | 57 | 113 | 0.1 | 59 | 35 | 63 |
| 005 | F | 1.7 | 58 | 55 | 20 | 57 | 111 | 58 | 21 | 57 | 112 | 4.8 | 52 | 42 | 121 |
| 006 | M | 1.8 | 55 | 76 | 25 | 58 | 111 | 78 | 25 | 57 | 109 | 2.7 | 0 | 42 | 173 |
| 007 | F | 1.7 | 60 | 73 | 24 | 57 | 108 | 74 | 25 | 57 | 114 | 1.6 | 59 | 35 | 109 |
| 008 | M | 1.7 | 59 | 75 | 26 | 57 | 106 | 72 | 25 | 57 | 115 | 3.5 | 59 | 42 | 99 |
| 009 | F | 1.7 | 29 | 85 | 30 | 61 | 110 | 90 | 32 | 57 | 113 | 5.7 | 0 | 35 | 485 |
| 010 | F | 1.7 | 56 | 76 | 28 | 57 | 93 | 77 | 28 | 58 | 151 | 1.2 | 143 | 35 | 74 |
| 011 | M | 1.9 | 52 | 104 | 29 | 57 | 109 | 102 | 29 | 57 | 111 | 1.9 | 10 | 35 | 197 |
| 012 | M | 1.7 | 27 | 75 | 25 | 57 | 110 | 75 | 25 | 57 | 113 | 0.4 | 52 | 35 | 116 |
| 013 | F | 1.7 | 54 | 52 | 19 | 57 | 111 | 51 | 19 | 57 | 108 | 2.5 | 80 | 40 | 142 |
| 014 | M | 1.8 | 32 | 88 | 28 | 59 | 95 | 90 | 28 | 57 | 104 | 2.3 | 55 | 35 | 706 |
| 015 | M | 1.7 | 49 | 90 | 31 | 57 | 120 | 87 | 30 | 58 | 116 | 3.6 | 120 | 35 | 41 |
| 016 | M | 1.8 | 39 | 84 | 25 | 57 | 116 | 84 | 25 | 57 | 107 | 0.2 | 66 | 42 | 42 |
| 017 | F | 1.7 | 27 | 59 | 20 | 57 | 119 | 58 | 20 | 57 | 118 | 1.5 | 106 | 35 | 319 |
| 018 | M | 1.8 | 31 | 80 | 24 | 57 | 106 | 77 | 23 | 57 | 116 | 4.3 | 50 | 35 | 102 |
| 019 | F | 1.6 | 25 | 60 | 23 | 57 | 98 | 61 | 23 | 57 | 118 | 0.8 | 97 | 30 | 277 |
| 020 | M | 1.8 | 31 | 70 | 22 | 57 | 107 | 71 | 22 | 57 | 107 | 1.6 | 48 | 35 | 317 |
| 021 | F | 1.7 | 42 | 64 | 22 | 57 | 99 | 64 | 22 | 57 | 111 | 0.6 | 163 | 34 | 269 |
| 022 | M | 1.8 | 19 | 83 | 26 | 57 | 108 | 85 | 26 | 57 | 114 | 1.6 | 57 | 35 | 88 |
| 023 | F | 1.8 | 22 | 68 | 22 | 57 | 119 | 69 | 22 | 57 | 116 | 1.6 | 73 | 36 | 101 |
| 024 | F | 1.6 | 25 | 59 | 24 | 57 | 111 | 57 | 20 | 57 | 115 | 3.6 | 69 | 36 | 95 |
| 025 | M | 1.8 | 26 | 77 | 25 | 57 | 118 | 77 | 24 | 57 | 103 | 0.4 | 73 | 35 | 36 |
| 026 | M | 1.9 | 25 | 94 | 26 | 57 | 110 | 94 | 25 | 57 | 125 | 0.5 | 45 | 42 | 324 |
| 027 | F | 1.7 | 35 | 65 | 22 | 57 | 115 | 67 | 23 | 57 | 114 | 3.5 | 41 | 33 | 129 |
| 028 | F | 1.7 | 26 | 61 | 21 | 57 | 110 | 62 | 22 | 57 | 122 | 1.1 | 70 | 49 | 331 |
| 029 | F | 1.7 | 33 | 53 | 19 | 57 | 108 | 53 | 19 | 57 | 108 | 0.4 | 57 | 35 | 316 |
| 030 | F | 1.9 | 21 | 91 | 25 | 57 | 111 | 92 | 25 | 57 | 112 | 1.1 | 56 | 46 | 22 |
| 031 | M | 1.9 | 32 | 68 | 19 | 57 | 115 | 69 | 19 | 57 | 94 | 1.8 | 77 | 33 | 284 |
| 032 | M | 1.8 | 52 | 88 | 26 | 57 | 118 | 86 | 26 | 65 | 102 | 1.5 | 27 | 35 | 68 |
| 033 | F | 1.7 | 50 | 60 | 20 | 57 | 99 | 60 | 20 | 57 | 113 | 0.2 | 0 | 35 | 83 |
| 034 | M | 1.8 | 25 | 89 | 26 | 57 | 114 | 91 | 27 | 57 | 106 | 2.7 | 28 | 36 | 118 |
| 035 | M | 1.7 | 31 | 85 | 28 | 57 | 105 | 86 | 28 | 57 | 106 | 1.8 | 41 | 36 | 255 |
| 036 | M | 1.8 | 59 | 85 | 25 | 57 | 124 | 85 | 25 | 57 | 118 | 0.2 | 45 | 48 | 35 |
| 037 | F | 1.7 | 30 | 68 | 24 | 57 | 111 | 68 | 24 | 57 | 105 | 0.0 | 38 | 49 | 289 |
| 038 | M | 1.8 | 53 | 81 | 24 | 57 | 119 | 81 | 24 | 57 | 109 | 0.6 | 35 | 41 | 244 |
| 039 | M | 1.8 | 27 | 75 | 24 | 57 | 112 | 75 | 24 | 57 | 99 | 0.1 | 45 | 22 | 35 |
| 040 | F | 1.7 | 29 | 67 | 23 | 57 | 103 | 69 | 24 | 57 | 110 | 2.1 | 35 | 33 | 274 |
| 041 | F | 1.7 | 23 | 73 | 24 | 57 | 102 | 74 | 24 | 57 | 110 | 1.5 | 30 | 41 | 283 |
| 042 | F | 1.8 | 50 | 74 | 24 | 57 | 99 | 75 | 24 | 57 | 109 | 2.0 | 42 | 46 | 264 |
| 043 | F | 1.7 | 25 | 63 | 22 | 57 | 106 | 63 | 22 | 57 | 115 | 0.3 | 41 | 34 | 219 |
| 044 | M | 1.9 | 34 | 113 | 31 | 58 | 103 | 115 | 32 | 57 | 107 | 2.1 | 35 | 49 | 90 |
| 045 | F | 1.6 | 65 | 56 | 22 | 57 | 112 | 55 | 22 | 57 | 113 | 1.3 | 29 | 47 | 226 |
| 046 | F | 1.7 | 65 | 49 | 18 | 60 | 117 | 51 | 19 | 57 | 109 | 3.0 | 29 | 33 | 240 |
| 047 | M | 1.8 | 33 | 95 | 28 | 57 | 110 | 94 | 28 | 57 | 108 | 1.3 | 22 | 40 | 36 |
| 048 | F | 1.7 | 24 | 67 | 25 | 57 | 110 | 70 | 26 | 57 | 107 | 3.7 | 35 | 30 | 86 |

**Table S2.** Selected reports of the normative values for CT (HU (mean ± STD)) and PET (SUV (mean ± STD)) for all 135 segmented regions, in addition to the CT-based volume estimates ([ml] (mean ± STD)), averaged across all 48 healthy controls. FOV – field-of-view of the PET/CT system.

| **Organ** | **HU** | | | | | | **SUV** | | | | | | **Volume [ml]** | | | | | |
| --- | --- | --- | --- | --- | --- | --- | --- | --- | --- | --- | --- | --- | --- | --- | --- | --- | --- | --- |
|  | Test | | | Retest | | | Test | | | Retest | | | Test | | | Retest | | |
| skeletal muscle | 42 | ± | 7 | 42 | ± | 7 | 0.6 | ± | 0.1 | 0.6 | ± | 0.1 | 816 | ± | 217 | 818 | ± | 207 |
| subcutaneous fat | -80 | ± | 10 | -80 | ± | 10 | 0.4 | ± | 0.1 | 0.4 | ± | 0.1 | 795 | ± | 455 | 797 | ± | 467 |
| visceral fat | -68 | ± | 11 | -69 | ± | 11 | 0.9 | ± | 0.2 | 0.8 | ± | 0.2 | 315 | ± | 228 | 309 | ± | 223 |
| heart myocardium | 32 | ± | 9 | 33 | ± | 9 | 3.8 | ± | 2.4 | 4.0 | ± | 3.1 | 116 | ± | 28 | 118 | ± | 28 |
| heart atrium left | 43 | ± | 4 | 42 | ± | 4 | 2.1 | ± | 0.3 | 2.1 | ± | 0.3 | 61 | ± | 15 | 60 | ± | 14 |
| heart atrium right | 31 | ± | 9 | 31 | ± | 11 | 1.9 | ± | 0.2 | 1.9 | ± | 0.2 | 89 | ± | 21 | 90 | ± | 19 |
| heart ventricle left | 42 | ± | 3 | 42 | ± | 3 | 3.1 | ± | 1.5 | 3.1 | ± | 1.5 | 117 | ± | 29 | 119 | ± | 29 |
| heart ventricle right | 40 | ± | 4 | 40 | ± | 4 | 2.1 | ± | 0.5 | 2.1 | ± | 0.6 | 162 | ± | 38 | 164 | ± | 35 |
| aorta | 40 | ± | 3 | 41 | ± | 3 | 1.9 | ± | 0.2 | 1.9 | ± | 0.2 | 180 | ± | 66 | 178 | ± | 65 |
| iliac artery left | 43 | ± | 4 | 42 | ± | 4 | 1.6 | ± | 0.2 | 1.7 | ± | 0.3 | 14 | ± | 6 | 14 | ± | 6 |
| iliac artery right | 44 | ± | 3 | 44 | ± | 3 | 1.9 | ± | 0.2 | 1.9 | ± | 0.3 | 16 | ± | 6 | 16 | ± | 6 |
| iliac vena left | 43 | ± | 4 | 43 | ± | 5 | 1.8 | ± | 0.4 | 1.8 | ± | 0.4 | 33 | ± | 9 | 33 | ± | 9 |
| iliac vena right | 44 | ± | 4 | 44 | ± | 4 | 2.1 | ± | 0.5 | 2.0 | ± | 0.5 | 25 | ± | 8 | 25 | ± | 7 |
| inferior vena cava | 42 | ± | 4 | 43 | ± | 6 | 1.9 | ± | 0.2 | 1.9 | ± | 0.2 | 61 | ± | 24 | 57 | ± | 18 |
| portal splenic vein | 44 | ± | 7 | 45 | ± | 7 | 1.9 | ± | 0.3 | 1.8 | ± | 0.2 | 5 | ± | 5 | 5 | ± | 5 |
| pulmonary artery | 41 | ± | 3 | 41 | ± | 4 | 1.9 | ± | 0.2 | 1.9 | ± | 0.2 | 46 | ± | 12 | 47 | ± | 13 |
| colon | -199 | ± | 98 | -207 | ± | 108 | 1.8 | ± | 1.0 | 1.4 | ± | 0.6 | 839 | ± | 347 | 894 | ± | 306 |
| duodenum | -55 | ± | 97 | -55 | ± | 104 | 1.8 | ± | 0.4 | 1.7 | ± | 0.3 | 42 | ± | 18 | 44 | ± | 18 |
| esophagus | 0 | ± | 40 | -3 | ± | 53 | 1.8 | ± | 0.2 | 1.8 | ± | 0.2 | 29 | ± | 7 | 29 | ± | 7 |
| small bowel | -55 | ± | 74 | -78 | ± | 109 | 3.0 | ± | 1.5 | 2.5 | ± | 1.2 | 620 | ± | 239 | 652 | ± | 228 |
| autochthon left | 46 | ± | 7 | 46 | ± | 8 | 0.7 | ± | 0.1 | 0.6 | ± | 0.1 | 594 | ± | 152 | 593 | ± | 152 |
| autochthon right | 45 | ± | 8 | 46 | ± | 9 | 0.7 | ± | 0.1 | 0.7 | ± | 0.1 | 587 | ± | 153 | 587 | ± | 152 |
| gluteus maximus left | 43 | ± | 8 | 43 | ± | 8 | 0.6 | ± | 0.1 | 0.6 | ± | 0.1 | 667 | ± | 165 | 677 | ± | 170 |
| gluteus maximus right | 42 | ± | 8 | 43 | ± | 8 | 0.6 | ± | 0.1 | 0.6 | ± | 0.1 | 686 | ± | 162 | 697 | ± | 169 |
| gluteus medius left | 46 | ± | 5 | 45 | ± | 5 | 0.6 | ± | 0.1 | 0.6 | ± | 0.1 | 268 | ± | 61 | 268 | ± | 60 |
| gluteus medius right | 46 | ± | 5 | 46 | ± | 5 | 0.7 | ± | 0.1 | 0.7 | ± | 0.1 | 268 | ± | 59 | 268 | ± | 58 |
| gluteus minimus left | 47 | ± | 7 | 47 | ± | 7 | 0.7 | ± | 0.1 | 0.7 | ± | 0.1 | 62 | ± | 13 | 62 | ± | 13 |
| gluteus minimus right | 47 | ± | 8 | 47 | ± | 8 | 0.7 | ± | 0.1 | 0.7 | ± | 0.1 | 69 | ± | 16 | 69 | ± | 15 |
| iliopsoas left | 55 | ± | 3 | 55 | ± | 4 | 0.7 | ± | 0.1 | 0.7 | ± | 0.1 | 370 | ± | 106 | 371 | ± | 107 |
| iliopsoas right | 53 | ± | 3 | 54 | ± | 4 | 0.8 | ± | 0.1 | 0.7 | ± | 0.1 | 360 | ± | 108 | 362 | ± | 107 |
| adrenal gland left | 21 | ± | 12 | 24 | ± | 14 | 1.8 | ± | 0.3 | 1.8 | ± | 0.3 | 2 | ± | 2 | 2 | ± | 2 |
| adrenal gland right | 8 | ± | 16 | 7 | ± | 15 | 1.9 | ± | 0.3 | 1.8 | ± | 0.3 | 2 | ± | 1 | 2 | ± | 1 |
| bladder | 20 | ± | 10 | 19 | ± | 10 | 35.4 | ± | 25.9 | 40.9 | ± | 34.1 | 79 | ± | 54 | 88 | ± | 48 |
| brain | 35 | ± | 2 | 35 | ± | 1 | 6.8 | ± | 1.0 | 6.8 | ± | 0.9 | 1377 | ± | 147 | 1378 | ± | 148 |
| gallbladder | 18 | ± | 5 | 18 | ± | 6 | 1.1 | ± | 0.4 | 1.0 | ± | 0.4 | 22 | ± | 11 | 23 | ± | 11 |
| kidney left | 25 | ± | 7 | 23 | ± | 10 | 2.9 | ± | 0.4 | 3.1 | ± | 0.6 | 129 | ± | 44 | 125 | ± | 42 |
| kidney right | 25 | ± | 8 | 25 | ± | 9 | 3.2 | ± | 0.7 | 3.4 | ± | 0.8 | 132 | ± | 39 | 129 | ± | 39 |
| liver | 54 | ± | 4 | 55 | ± | 4 | 2.3 | ± | 0.3 | 2.3 | ± | 0.2 | 1534 | ± | 283 | 1559 | ± | 267 |
| lung lower lobe left | -634 | ± | 73 | -621 | ± | 74 | 0.8 | ± | 0.2 | 0.8 | ± | 0.1 | 613 | ± | 153 | 594 | ± | 131 |
| lung lower lobe right | -654 | ± | 67 | -644 | ± | 67 | 0.8 | ± | 0.2 | 0.8 | ± | 0.2 | 683 | ± | 176 | 669 | ± | 157 |
| lung middle lobe right | -772 | ± | 44 | -769 | ± | 43 | 0.5 | ± | 0.1 | 0.5 | ± | 0.1 | 323 | ± | 80 | 323 | ± | 80 |
| lung upper lobe left | -731 | ± | 53 | -725 | ± | 54 | 0.6 | ± | 0.1 | 0.6 | ± | 0.1 | 783 | ± | 235 | 770 | ± | 226 |
| lung upper lobe right | -737 | ± | 54 | -731 | ± | 53 | 0.5 | ± | 0.1 | 0.5 | ± | 0.1 | 636 | ± | 179 | 627 | ± | 173 |
| pancreas | 43 | ± | 5 | 44 | ± | 5 | 1.6 | ± | 0.2 | 1.6 | ± | 0.2 | 79 | ± | 22 | 81 | ± | 22 |
| spleen | 37 | ± | 9 | 35 | ± | 12 | 1.8 | ± | 0.2 | 1.7 | ± | 0.2 | 186 | ± | 63 | 185 | ± | 65 |
| stomach | -156 | ± | 112 | -135 | ± | 102 | 1.4 | ± | 0.3 | 1.5 | ± | 0.2 | 231 | ± | 92 | 208 | ± | 55 |
| thyroid left | 73 | ± | 9 | 74 | ± | 10 | 1.3 | ± | 0.3 | 1.3 | ± | 0.4 | 6 | ± | 2 | 6 | ± | 2 |
| thyroid right | 74 | ± | 9 | 75 | ± | 10 | 1.4 | ± | 0.3 | 1.4 | ± | 0.4 | 6 | ± | 2 | 6 | ± | 2 |
| trachea | -837 | ± | 20 | -834 | ± | 21 | 0.8 | ± | 0.1 | 0.8 | ± | 0.1 | 32 | ± | 9 | 32 | ± | 9 |
| carpal left | 251 | ± | 44 | 247 | ± | 66 | 0.6 | ± | 0.2 | 0.6 | ± | 0.2 | 20 | ± | 9 | 20 | ± | 7 |
| carpal right | 250 | ± | 36 | 250 | ± | 39 | 0.6 | ± | 0.2 | 0.7 | ± | 0.2 | 20 | ± | 7 | 20 | ± | 6 |
| clavicle left | 368 | ± | 53 | 370 | ± | 54 | 0.8 | ± | 0.2 | 0.8 | ± | 0.1 | 31 | ± | 7 | 30 | ± | 7 |
| clavicle right | 370 | ± | 50 | 371 | ± | 53 | 0.8 | ± | 0.1 | 0.8 | ± | 0.1 | 31 | ± | 8 | 31 | ± | 8 |
| femur left | 410 | ± | 57 | 413 | ± | 48 | 0.6 | ± | 0.1 | 0.6 | ± | 0.1 | 251 | ± | 34 | 254 | ± | 31 |
| femur right | 405 | ± | 57 | 409 | ± | 48 | 0.6 | ± | 0.1 | 0.6 | ± | 0.1 | 252 | ± | 33 | 255 | ± | 30 |
| fibula left | not in FOV | | | | | | | | | | | | | | | | | |
| fibula right | not in FOV | | | | | | | | | | | | | | | | | |
| fingers left | 328 | ± | 43 | 317 | ± | 43 | 0.5 | ± | 0.2 | 0.6 | ± | 0.2 | 9 | ± | 6 | 8 | ± | 5 |
| fingers right | 331 | ± | 44 | 328 | ± | 52 | 0.7 | ± | 0.3 | 0.7 | ± | 0.2 | 12 | ± | 5 | 11 | ± | 5 |
| humerus left | 359 | ± | 38 | 350 | ± | 36 | 0.5 | ± | 0.1 | 0.5 | ± | 0.1 | 190 | ± | 38 | 189 | ± | 39 |
| humerus right | 362 | ± | 38 | 360 | ± | 38 | 0.5 | ± | 0.1 | 0.5 | ± | 0.1 | 195 | ± | 40 | 191 | ± | 39 |
| metacarpal left | 307 | ± | 53 | 305 | ± | 40 | 0.6 | ± | 0.2 | 0.6 | ± | 0.2 | 25 | ± | 13 | 25 | ± | 10 |
| metacarpal right | 306 | ± | 46 | 313 | ± | 60 | 0.6 | ± | 0.2 | 0.7 | ± | 0.2 | 30 | ± | 10 | 30 | ± | 9 |
| metatarsal left | not in FOV | | | | | | | | | | | | | | | | | |
| metatarsal right | not in FOV | | | | | | | | | | | | | | | | | |
| patella left | not in FOV | | | | | | | | | | | | | | | | | |
| patella right | not in FOV | | | | | | | | | | | | | | | | | |
| radius left | 399 | ± | 58 | 382 | ± | 60 | 0.6 | ± | 0.1 | 0.6 | ± | 0.1 | 44 | ± | 13 | 45 | ± | 14 |
| radius right | 402 | ± | 60 | 383 | ± | 66 | 0.5 | ± | 0.1 | 0.6 | ± | 0.1 | 49 | ± | 13 | 44 | ± | 12 |
| scapula left | 322 | ± | 38 | 322 | ± | 38 | 0.8 | ± | 0.1 | 0.8 | ± | 0.1 | 105 | ± | 25 | 105 | ± | 25 |
| scapula right | 327 | ± | 37 | 327 | ± | 37 | 0.8 | ± | 0.1 | 0.8 | ± | 0.1 | 107 | ± | 26 | 107 | ± | 25 |
| skull | 569 | ± | 54 | 569 | ± | 55 | 1.8 | ± | 0.4 | 1.8 | ± | 0.3 | 801 | ± | 107 | 802 | ± | 106 |
| tarsal left | not in FOV | | | | | | | | | | | | | | | | | |
| tarsal right | not in FOV | | | | | | | | | | | | | | | | | |
| tibia left | not in FOV | | | | | | | | | | | | | | | | | |
| tibia right | not in FOV | | | | | | | | | | | | | | | | | |
| toes left | not in FOV | | | | | | | | | | | | | | | | | |
| toes right | not in FOV | | | | | | | | | | | | | | | | | |
| ulna left | 368 | ± | 58 | 343 | ± | 60 | 0.5 | ± | 0.1 | 0.5 | ± | 0.1 | 57 | ± | 16 | 57 | ± | 15 |
| ulna right | 360 | ± | 68 | 339 | ± | 68 | 0.5 | ± | 0.1 | 0.5 | ± | 0.1 | 60 | ± | 14 | 55 | ± | 15 |
| rib left 1 | 297 | ± | 51 | 297 | ± | 51 | 0.9 | ± | 0.2 | 0.9 | ± | 0.2 | 10 | ± | 3 | 10 | ± | 3 |
| rib left 2 | 279 | ± | 59 | 278 | ± | 58 | 0.9 | ± | 0.1 | 0.9 | ± | 0.1 | 13 | ± | 3 | 13 | ± | 3 |
| rib left 3 | 285 | ± | 66 | 285 | ± | 67 | 0.9 | ± | 0.1 | 0.9 | ± | 0.1 | 15 | ± | 4 | 15 | ± | 4 |
| rib left 4 | 292 | ± | 63 | 294 | ± | 64 | 1.0 | ± | 0.1 | 1.0 | ± | 0.1 | 20 | ± | 5 | 20 | ± | 5 |
| rib left 5 | 293 | ± | 66 | 294 | ± | 67 | 1.0 | ± | 0.2 | 1.0 | ± | 0.1 | 22 | ± | 5 | 22 | ± | 5 |
| rib left 6 | 304 | ± | 60 | 305 | ± | 59 | 1.0 | ± | 0.1 | 1.0 | ± | 0.1 | 25 | ± | 6 | 25 | ± | 6 |
| rib left 7 | 314 | ± | 59 | 315 | ± | 59 | 1.1 | ± | 0.2 | 1.0 | ± | 0.1 | 26 | ± | 6 | 26 | ± | 6 |
| rib left 8 | 318 | ± | 62 | 318 | ± | 63 | 1.1 | ± | 0.1 | 1.0 | ± | 0.1 | 22 | ± | 5 | 22 | ± | 5 |
| rib left 9 | 316 | ± | 62 | 315 | ± | 63 | 1.1 | ± | 0.1 | 1.1 | ± | 0.1 | 20 | ± | 4 | 20 | ± | 5 |
| rib left 10 | 333 | ± | 59 | 333 | ± | 60 | 1.0 | ± | 0.1 | 1.0 | ± | 0.1 | 16 | ± | 4 | 16 | ± | 4 |
| rib left 11 | 346 | ± | 68 | 346 | ± | 68 | 1.0 | ± | 0.1 | 1.0 | ± | 0.1 | 11 | ± | 3 | 11 | ± | 3 |
| rib left 12 | 319 | ± | 65 | 320 | ± | 66 | 1.1 | ± | 0.2 | 1.1 | ± | 0.2 | 5 | ± | 3 | 5 | ± | 3 |
| rib left 13 | not present in the cohort | | | | | | | | | | | | | | | | | |
| rib right 1 | 307 | ± | 49 | 305 | ± | 49 | 1.0 | ± | 0.2 | 1.0 | ± | 0.2 | 10 | ± | 3 | 10 | ± | 2 |
| rib right 2 | 290 | ± | 61 | 289 | ± | 60 | 0.9 | ± | 0.1 | 0.9 | ± | 0.1 | 13 | ± | 3 | 13 | ± | 3 |
| rib right 3 | 287 | ± | 66 | 288 | ± | 66 | 0.9 | ± | 0.1 | 0.9 | ± | 0.1 | 15 | ± | 4 | 15 | ± | 4 |
| rib right 4 | 295 | ± | 66 | 295 | ± | 67 | 1.0 | ± | 0.1 | 1.0 | ± | 0.1 | 19 | ± | 5 | 19 | ± | 5 |
| rib right 5 | 295 | ± | 66 | 295 | ± | 68 | 1.0 | ± | 0.1 | 1.0 | ± | 0.1 | 22 | ± | 5 | 22 | ± | 5 |
| rib right 6 | 307 | ± | 65 | 308 | ± | 65 | 1.1 | ± | 0.2 | 1.1 | ± | 0.2 | 25 | ± | 6 | 25 | ± | 5 |
| rib right 7 | 314 | ± | 61 | 315 | ± | 60 | 1.1 | ± | 0.2 | 1.1 | ± | 0.2 | 25 | ± | 6 | 25 | ± | 6 |
| rib right 8 | 319 | ± | 62 | 320 | ± | 61 | 1.2 | ± | 0.2 | 1.1 | ± | 0.2 | 22 | ± | 5 | 22 | ± | 5 |
| rib right 9 | 319 | ± | 62 | 320 | ± | 61 | 1.2 | ± | 0.1 | 1.1 | ± | 0.1 | 20 | ± | 4 | 20 | ± | 4 |
| rib right 10 | 338 | ± | 61 | 338 | ± | 60 | 1.2 | ± | 0.1 | 1.1 | ± | 0.2 | 15 | ± | 4 | 15 | ± | 3 |
| rib right 11 | 355 | ± | 68 | 355 | ± | 67 | 1.1 | ± | 0.2 | 1.1 | ± | 0.2 | 10 | ± | 3 | 10 | ± | 3 |
| rib right 12 | 320 | ± | 66 | 320 | ± | 67 | 1.0 | ± | 0.2 | 1.0 | ± | 0.2 | 4 | ± | 2 | 5 | ± | 2 |
| rib right 13 | not present in the cohort | | | | | | | | | | | | | | | | | |
| sternum | 158 | ± | 37 | 159 | ± | 37 | 1.2 | ± | 0.3 | 1.2 | ± | 0.3 | 60 | ± | 14 | 60 | ± | 15 |
| vertebra C1 | 420 | ± | 51 | 419 | ± | 50 | 1.3 | ± | 0.1 | 1.3 | ± | 0.1 | 15 | ± | 2 | 15 | ± | 2 |
| vertebra C2 | 395 | ± | 55 | 394 | ± | 55 | 1.3 | ± | 0.1 | 1.3 | ± | 0.1 | 19 | ± | 3 | 19 | ± | 3 |
| vertebra C3 | 380 | ± | 59 | 381 | ± | 59 | 1.3 | ± | 0.1 | 1.3 | ± | 0.1 | 13 | ± | 2 | 13 | ± | 2 |
| vertebra C4 | 405 | ± | 57 | 405 | ± | 56 | 1.3 | ± | 0.1 | 1.3 | ± | 0.1 | 13 | ± | 2 | 13 | ± | 2 |
| vertebra C5 | 389 | ± | 57 | 391 | ± | 54 | 1.3 | ± | 0.1 | 1.3 | ± | 0.1 | 13 | ± | 2 | 13 | ± | 2 |
| vertebra C6 | 352 | ± | 50 | 353 | ± | 49 | 1.3 | ± | 0.1 | 1.3 | ± | 0.1 | 15 | ± | 3 | 15 | ± | 2 |
| vertebra C7 | 300 | ± | 42 | 299 | ± | 41 | 1.3 | ± | 0.1 | 1.3 | ± | 0.1 | 19 | ± | 3 | 19 | ± | 3 |
| vertebra T1 | 276 | ± | 38 | 274 | ± | 36 | 1.4 | ± | 0.2 | 1.4 | ± | 0.2 | 24 | ± | 4 | 24 | ± | 4 |
| vertebra T2 | 270 | ± | 38 | 270 | ± | 38 | 1.4 | ± | 0.2 | 1.4 | ± | 0.2 | 23 | ± | 4 | 24 | ± | 4 |
| vertebra T3 | 273 | ± | 40 | 272 | ± | 38 | 1.4 | ± | 0.2 | 1.4 | ± | 0.2 | 22 | ± | 4 | 22 | ± | 4 |
| vertebra T4 | 269 | ± | 39 | 269 | ± | 39 | 1.5 | ± | 0.2 | 1.5 | ± | 0.2 | 23 | ± | 4 | 23 | ± | 4 |
| vertebra T5 | 266 | ± | 38 | 266 | ± | 38 | 1.5 | ± | 0.2 | 1.5 | ± | 0.2 | 25 | ± | 5 | 25 | ± | 5 |
| vertebra T6 | 265 | ± | 39 | 264 | ± | 39 | 1.5 | ± | 0.2 | 1.5 | ± | 0.2 | 27 | ± | 5 | 27 | ± | 5 |
| vertebra T7 | 261 | ± | 37 | 259 | ± | 36 | 1.5 | ± | 0.2 | 1.5 | ± | 0.2 | 29 | ± | 6 | 29 | ± | 6 |
| vertebra T8 | 259 | ± | 40 | 256 | ± | 38 | 1.5 | ± | 0.2 | 1.5 | ± | 0.2 | 31 | ± | 7 | 31 | ± | 6 |
| vertebra T9 | 260 | ± | 41 | 259 | ± | 39 | 1.5 | ± | 0.2 | 1.5 | ± | 0.2 | 34 | ± | 7 | 34 | ± | 7 |
| vertebra T10 | 262 | ± | 45 | 258 | ± | 41 | 1.6 | ± | 0.2 | 1.5 | ± | 0.2 | 37 | ± | 8 | 37 | ± | 8 |
| vertebra T11 | 252 | ± | 41 | 249 | ± | 40 | 1.6 | ± | 0.2 | 1.6 | ± | 0.2 | 40 | ± | 7 | 40 | ± | 8 |
| vertebra T12 | 250 | ± | 42 | 247 | ± | 41 | 1.6 | ± | 0.2 | 1.6 | ± | 0.2 | 45 | ± | 9 | 45 | ± | 9 |
| vertebra L1 | 263 | ± | 43 | 261 | ± | 43 | 1.6 | ± | 0.2 | 1.5 | ± | 0.2 | 52 | ± | 11 | 52 | ± | 10 |
| vertebra L2 | 277 | ± | 44 | 277 | ± | 45 | 1.5 | ± | 0.2 | 1.5 | ± | 0.2 | 56 | ± | 11 | 56 | ± | 10 |
| vertebra L3 | 279 | ± | 46 | 278 | ± | 46 | 1.5 | ± | 0.2 | 1.5 | ± | 0.2 | 62 | ± | 11 | 62 | ± | 11 |
| vertebra L4 | 279 | ± | 44 | 279 | ± | 45 | 1.5 | ± | 0.2 | 1.5 | ± | 0.2 | 63 | ± | 11 | 63 | ± | 11 |
| vertebra L5 | 282 | ± | 43 | 282 | ± | 44 | 1.5 | ± | 0.3 | 1.5 | ± | 0.2 | 62 | ± | 10 | 62 | ± | 11 |
| vertebra L6 | 306 | ± | 130 | 374 | ± | 105 | 1.5 | ± | 0.6 | 1.3 | ± | 0.3 | 5 | ± | 13 | 7 | ± | 15 |
| hip left | 309 | ± | 49 | 308 | ± | 48 | 1.1 | ± | 0.2 | 1.1 | ± | 0.2 | 368 | ± | 65 | 371 | ± | 69 |
| hip right | 311 | ± | 49 | 311 | ± | 50 | 1.2 | ± | 0.2 | 1.2 | ± | 0.2 | 366 | ± | 66 | 365 | ± | 67 |
| sacrum | 194 | ± | 39 | 195 | ± | 40 | 1.2 | ± | 0.3 | 1.3 | ± | 0.2 | 223 | ± | 31 | 223 | ± | 32 |

**Table S3**. Selected reports of the normative values for CT (HU (mean ± STD)) and PET (SUV (mean ± STD)) for all 135 segmented regions, in addition to the CT-based volume estimates ([ml] (mean ± STD)), averaged across all 23 male healthy controls. FOV – field-of-view of the PET/CT system.

| **Organ** | **HU** | | | | | | **SUV** | | | | | | **Volume [ml]** | | | | | |
| --- | --- | --- | --- | --- | --- | --- | --- | --- | --- | --- | --- | --- | --- | --- | --- | --- | --- | --- |
|  | Test | | | Retest | | | Test | | | Retest | | | Test | | | Retest | | |
| skeletal muscle | 44 | ± | 4 | 45 | ± | 4 | 0.6 | ± | 0.1 | 0.6 | ± | 0.0 | 1004 | ± | 112 | 994 | ± | 103 |
| subcutaneous fat | -83 | ± | 8 | -81 | ± | 7 | 0.4 | ± | 0.1 | 0.4 | ± | 0.1 | 835 | ± | 383 | 827 | ± | 369 |
| visceral fat | -74 | ± | 8 | -74 | ± | 8 | 0.8 | ± | 0.2 | 0.8 | ± | 0.2 | 440 | ± | 189 | 428 | ± | 180 |
| heart myocardium | 32 | ± | 11 | 35 | ± | 9 | 3.8 | ± | 2.2 | 3.9 | ± | 2.7 | 136 | ± | 26 | 139 | ± | 23 |
| heart atrium left | 45 | ± | 3 | 45 | ± | 4 | 2.1 | ± | 0.3 | 2.1 | ± | 0.2 | 71 | ± | 14 | 70 | ± | 11 |
| heart atrium right | 34 | ± | 9 | 35 | ± | 10 | 2.0 | ± | 0.3 | 2.0 | ± | 0.2 | 103 | ± | 19 | 103 | ± | 14 |
| heart ventricle left | 43 | ± | 3 | 44 | ± | 3 | 3.2 | ± | 1.4 | 3.1 | ± | 1.3 | 135 | ± | 29 | 139 | ± | 26 |
| heart ventricle right | 42 | ± | 4 | 42 | ± | 4 | 2.1 | ± | 0.5 | 2.1 | ± | 0.4 | 193 | ± | 28 | 193 | ± | 23 |
| aorta | 43 | ± | 2 | 42 | ± | 3 | 2.0 | ± | 0.2 | 2.0 | ± | 0.2 | 214 | ± | 66 | 210 | ± | 65 |
| iliac artery left | 44 | ± | 3 | 45 | ± | 3 | 1.6 | ± | 0.3 | 1.6 | ± | 0.3 | 18 | ± | 6 | 18 | ± | 7 |
| iliac artery right | 46 | ± | 3 | 46 | ± | 2 | 1.9 | ± | 0.3 | 1.9 | ± | 0.3 | 20 | ± | 6 | 20 | ± | 5 |
| iliac vena left | 45 | ± | 3 | 46 | ± | 3 | 1.6 | ± | 0.3 | 1.7 | ± | 0.3 | 38 | ± | 8 | 38 | ± | 8 |
| iliac vena right | 47 | ± | 3 | 47 | ± | 4 | 2.0 | ± | 0.4 | 2.1 | ± | 0.5 | 31 | ± | 6 | 30 | ± | 6 |
| inferior vena cava | 44 | ± | 4 | 46 | ± | 7 | 1.9 | ± | 0.2 | 1.9 | ± | 0.2 | 75 | ± | 22 | 69 | ± | 14 |
| portal splenic vein | 44 | ± | 5 | 46 | ± | 7 | 1.8 | ± | 0.3 | 1.8 | ± | 0.2 | 7 | ± | 5 | 7 | ± | 6 |
| pulmonary artery | 42 | ± | 3 | 43 | ± | 4 | 1.9 | ± | 0.2 | 1.9 | ± | 0.2 | 52 | ± | 11 | 55 | ± | 12 |
| colon | -199 | ± | 119 | -224 | ± | 113 | 1.8 | ± | 1.0 | 1.5 | ± | 0.7 | 906 | ± | 409 | 912 | ± | 297 |
| duodenum | -13 | ± | 64 | -8 | ± | 49 | 1.8 | ± | 0.4 | 1.7 | ± | 0.2 | 51 | ± | 14 | 53 | ± | 12 |
| esophagus | -9 | ± | 48 | -12 | ± | 67 | 1.7 | ± | 0.2 | 1.7 | ± | 0.2 | 34 | ± | 5 | 34 | ± | 4 |
| small bowel | -50 | ± | 74 | -56 | ± | 85 | 2.5 | ± | 1.7 | 2.3 | ± | 1.2 | 771 | ± | 181 | 787 | ± | 145 |
| autochthon left | 48 | ± | 4 | 50 | ± | 5 | 0.6 | ± | 0.1 | 0.6 | ± | 0.1 | 713 | ± | 99 | 712 | ± | 101 |
| autochthon right | 48 | ± | 3 | 50 | ± | 5 | 0.7 | ± | 0.1 | 0.6 | ± | 0.1 | 710 | ± | 95 | 708 | ± | 95 |
| gluteus maximus left | 45 | ± | 5 | 46 | ± | 5 | 0.6 | ± | 0.1 | 0.6 | ± | 0.1 | 787 | ± | 132 | 805 | ± | 126 |
| gluteus maximus right | 45 | ± | 4 | 46 | ± | 5 | 0.6 | ± | 0.1 | 0.6 | ± | 0.1 | 803 | ± | 127 | 821 | ± | 124 |
| gluteus medius left | 46 | ± | 5 | 47 | ± | 4 | 0.6 | ± | 0.1 | 0.6 | ± | 0.1 | 311 | ± | 50 | 310 | ± | 49 |
| gluteus medius right | 46 | ± | 5 | 47 | ± | 4 | 0.6 | ± | 0.1 | 0.7 | ± | 0.1 | 309 | ± | 47 | 308 | ± | 45 |
| gluteus minimus left | 49 | ± | 4 | 50 | ± | 3 | 0.7 | ± | 0.1 | 0.7 | ± | 0.1 | 71 | ± | 11 | 71 | ± | 11 |
| gluteus minimus right | 50 | ± | 4 | 51 | ± | 4 | 0.7 | ± | 0.1 | 0.7 | ± | 0.1 | 80 | ± | 12 | 80 | ± | 12 |
| iliopsoas left | 55 | ± | 2 | 56 | ± | 4 | 0.6 | ± | 0.1 | 0.6 | ± | 0.1 | 457 | ± | 60 | 458 | ± | 65 |
| iliopsoas right | 54 | ± | 3 | 55 | ± | 4 | 0.7 | ± | 0.1 | 0.7 | ± | 0.1 | 448 | ± | 69 | 447 | ± | 72 |
| adrenal gland left | 16 | ± | 10 | 20 | ± | 11 | 1.8 | ± | 0.3 | 1.7 | ± | 0.3 | 4 | ± | 1 | 3 | ± | 1 |
| adrenal gland right | 0 | ± | 14 | 0 | ± | 16 | 1.9 | ± | 0.2 | 1.9 | ± | 0.3 | 3 | ± | 1 | 3 | ± | 1 |
| bladder | 21 | ± | 8 | 20 | ± | 11 | 42.3 | ± | 28.2 | 45.2 | ± | 35.6 | 91 | ± | 52 | 100 | ± | 50 |
| brain | 35 | ± | 2 | 34 | ± | 1 | 6.8 | ± | 1.1 | 6.8 | ± | 1.1 | 1463 | ± | 124 | 1465 | ± | 124 |
| gallbladder | 18 | ± | 4 | 19 | ± | 6 | 1.1 | ± | 0.3 | 1.0 | ± | 0.4 | 27 | ± | 12 | 27 | ± | 12 |
| kidney left | 21 | ± | 8 | 18 | ± | 11 | 2.9 | ± | 0.4 | 3.0 | ± | 0.4 | 155 | ± | 34 | 151 | ± | 27 |
| kidney right | 21 | ± | 10 | 20 | ± | 10 | 3.1 | ± | 0.5 | 3.2 | ± | 0.5 | 154 | ± | 29 | 154 | ± | 27 |
| liver | 53 | ± | 4 | 54 | ± | 4 | 2.4 | ± | 0.2 | 2.3 | ± | 0.2 | 1667 | ± | 220 | 1680 | ± | 228 |
| lung lower lobe left | -617 | ± | 74 | -600 | ± | 71 | 0.8 | ± | 0.2 | 0.8 | ± | 0.1 | 651 | ± | 170 | 628 | ± | 128 |
| lung lower lobe right | -638 | ± | 70 | -623 | ± | 64 | 0.8 | ± | 0.2 | 0.8 | ± | 0.2 | 721 | ± | 198 | 702 | ± | 161 |
| lung middle lobe right | -771 | ± | 45 | -767 | ± | 43 | 0.5 | ± | 0.1 | 0.5 | ± | 0.1 | 364 | ± | 76 | 365 | ± | 75 |
| lung upper lobe left | -724 | ± | 57 | -717 | ± | 53 | 0.6 | ± | 0.1 | 0.6 | ± | 0.1 | 879 | ± | 232 | 858 | ± | 204 |
| lung upper lobe right | -728 | ± | 61 | -722 | ± | 54 | 0.5 | ± | 0.1 | 0.5 | ± | 0.1 | 717 | ± | 184 | 701 | ± | 165 |
| pancreas | 42 | ± | 6 | 43 | ± | 6 | 1.6 | ± | 0.2 | 1.6 | ± | 0.2 | 94 | ± | 16 | 94 | ± | 15 |
| spleen | 33 | ± | 11 | 31 | ± | 16 | 1.8 | ± | 0.2 | 1.7 | ± | 0.2 | 209 | ± | 55 | 211 | ± | 58 |
| stomach | -140 | ± | 88 | -141 | ± | 70 | 1.4 | ± | 0.3 | 1.5 | ± | 0.2 | 240 | ± | 70 | 225 | ± | 40 |
| thyroid left | 73 | ± | 8 | 77 | ± | 9 | 1.4 | ± | 0.4 | 1.4 | ± | 0.4 | 6 | ± | 2 | 6 | ± | 2 |
| thyroid right | 74 | ± | 8 | 77 | ± | 8 | 1.5 | ± | 0.4 | 1.4 | ± | 0.4 | 6 | ± | 2 | 6 | ± | 2 |
| trachea | -836 | ± | 24 | -835 | ± | 24 | 0.7 | ± | 0.1 | 0.7 | ± | 0.1 | 38 | ± | 8 | 38 | ± | 7 |
| carpal left | 250 | ± | 35 | 237 | ± | 39 | 0.6 | ± | 0.3 | 0.7 | ± | 0.2 | 22 | ± | 7 | 22 | ± | 6 |
| carpal right | 262 | ± | 27 | 246 | ± | 48 | 0.6 | ± | 0.2 | 0.7 | ± | 0.2 | 24 | ± | 5 | 21 | ± | 7 |
| clavicle left | 365 | ± | 46 | 367 | ± | 49 | 0.8 | ± | 0.1 | 0.8 | ± | 0.1 | 36 | ± | 6 | 36 | ± | 6 |
| clavicle right | 365 | ± | 44 | 366 | ± | 49 | 0.8 | ± | 0.1 | 0.8 | ± | 0.1 | 36 | ± | 5 | 36 | ± | 6 |
| femur left | 387 | ± | 58 | 396 | ± | 45 | 0.6 | ± | 0.1 | 0.6 | ± | 0.1 | 261 | ± | 38 | 269 | ± | 29 |
| femur right | 380 | ± | 57 | 389 | ± | 44 | 0.6 | ± | 0.1 | 0.6 | ± | 0.1 | 263 | ± | 37 | 271 | ± | 25 |
| fibula left | not in FOV | | | | | | | | | | | | | | | | | |
| fibula right | not in FOV | | | | | | | | | | | | | | | | | |
| fingers left | 340 | ± | 41 | 314 | ± | 41 | 0.5 | ± | 0.3 | 0.6 | ± | 0.2 | 10 | ± | 6 | 8 | ± | 5 |
| fingers right | 343 | ± | 42 | 334 | ± | 47 | 0.6 | ± | 0.3 | 0.8 | ± | 0.2 | 14 | ± | 5 | 11 | ± | 5 |
| humerus left | 371 | ± | 38 | 362 | ± | 32 | 0.5 | ± | 0.1 | 0.5 | ± | 0.1 | 220 | ± | 23 | 218 | ± | 25 |
| humerus right | 372 | ± | 37 | 368 | ± | 39 | 0.5 | ± | 0.1 | 0.5 | ± | 0.1 | 226 | ± | 26 | 219 | ± | 26 |
| metacarpal left | 314 | ± | 48 | 304 | ± | 41 | 0.6 | ± | 0.2 | 0.7 | ± | 0.2 | 31 | ± | 10 | 29 | ± | 9 |
| metacarpal right | 313 | ± | 36 | 307 | ± | 54 | 0.6 | ± | 0.2 | 0.7 | ± | 0.2 | 36 | ± | 5 | 33 | ± | 10 |
| metatarsal left | not in FOV | | | | | | | | | | | | | | | | | |
| metatarsal right | not in FOV | | | | | | | | | | | | | | | | | |
| patella left | not in FOV | | | | | | | | | | | | | | | | | |
| patella right | not in FOV | | | | | | | | | | | | | | | | | |
| radius left | 405 | ± | 57 | 367 | ± | 65 | 0.6 | ± | 0.1 | 0.6 | ± | 0.1 | 53 | ± | 9 | 50 | ± | 10 |
| radius right | 401 | ± | 61 | 364 | ± | 70 | 0.5 | ± | 0.1 | 0.6 | ± | 0.1 | 58 | ± | 8 | 48 | ± | 13 |
| scapula left | 328 | ± | 32 | 328 | ± | 32 | 0.8 | ± | 0.1 | 0.8 | ± | 0.1 | 125 | ± | 15 | 125 | ± | 15 |
| scapula right | 332 | ± | 32 | 332 | ± | 31 | 0.8 | ± | 0.1 | 0.8 | ± | 0.1 | 128 | ± | 16 | 127 | ± | 16 |
| skull | 554 | ± | 52 | 553 | ± | 52 | 1.8 | ± | 0.4 | 1.8 | ± | 0.3 | 856 | ± | 87 | 857 | ± | 86 |
| tarsal left | not in FOV | | | | | | | | | | | | | | | | | |
| tarsal right | not in FOV | | | | | | | | | | | | | | | | | |
| tibia left | not in FOV | | | | | | | | | | | | | | | | | |
| tibia right | not in FOV | | | | | | | | | | | | | | | | | |
| toes left | not in FOV | | | | | | | | | | | | | | | | | |
| toes right | not in FOV | | | | | | | | | | | | | | | | | |
| ulna left | 372 | ± | 61 | 333 | ± | 62 | 0.5 | ± | 0.1 | 0.5 | ± | 0.1 | 68 | ± | 11 | 66 | ± | 10 |
| ulna right | 351 | ± | 71 | 315 | ± | 69 | 0.5 | ± | 0.1 | 0.5 | ± | 0.1 | 70 | ± | 10 | 61 | ± | 15 |
| rib left 1 | 296 | ± | 47 | 299 | ± | 45 | 0.9 | ± | 0.1 | 0.9 | ± | 0.1 | 12 | ± | 3 | 12 | ± | 2 |
| rib left 2 | 267 | ± | 48 | 266 | ± | 46 | 0.9 | ± | 0.1 | 0.9 | ± | 0.1 | 15 | ± | 3 | 15 | ± | 2 |
| rib left 3 | 272 | ± | 55 | 270 | ± | 53 | 1.0 | ± | 0.1 | 1.0 | ± | 0.1 | 18 | ± | 3 | 18 | ± | 3 |
| rib left 4 | 279 | ± | 53 | 280 | ± | 53 | 1.0 | ± | 0.1 | 1.0 | ± | 0.1 | 23 | ± | 4 | 24 | ± | 4 |
| rib left 5 | 278 | ± | 60 | 279 | ± | 60 | 1.0 | ± | 0.2 | 1.0 | ± | 0.1 | 26 | ± | 4 | 26 | ± | 4 |
| rib left 6 | 289 | ± | 54 | 290 | ± | 54 | 1.1 | ± | 0.2 | 1.1 | ± | 0.1 | 30 | ± | 5 | 30 | ± | 5 |
| rib left 7 | 299 | ± | 51 | 301 | ± | 51 | 1.1 | ± | 0.2 | 1.1 | ± | 0.1 | 31 | ± | 4 | 31 | ± | 4 |
| rib left 8 | 305 | ± | 55 | 305 | ± | 56 | 1.1 | ± | 0.1 | 1.1 | ± | 0.1 | 26 | ± | 4 | 26 | ± | 4 |
| rib left 9 | 307 | ± | 56 | 307 | ± | 58 | 1.1 | ± | 0.1 | 1.1 | ± | 0.1 | 24 | ± | 3 | 24 | ± | 3 |
| rib left 10 | 329 | ± | 57 | 329 | ± | 58 | 1.0 | ± | 0.1 | 1.0 | ± | 0.1 | 19 | ± | 3 | 19 | ± | 3 |
| rib left 11 | 340 | ± | 65 | 340 | ± | 66 | 1.0 | ± | 0.1 | 1.0 | ± | 0.1 | 13 | ± | 3 | 13 | ± | 3 |
| rib left 12 | 314 | ± | 61 | 313 | ± | 63 | 1.0 | ± | 0.2 | 1.0 | ± | 0.2 | 6 | ± | 3 | 6 | ± | 3 |
| rib left 13 | not present in the cohort | | | | | | | | | | | | | | | | | |
| rib right 1 | 303 | ± | 43 | 306 | ± | 41 | 1.0 | ± | 0.1 | 1.0 | ± | 0.1 | 11 | ± | 2 | 11 | ± | 2 |
| rib right 2 | 274 | ± | 49 | 273 | ± | 47 | 1.0 | ± | 0.1 | 1.0 | ± | 0.1 | 14 | ± | 3 | 14 | ± | 2 |
| rib right 3 | 272 | ± | 52 | 271 | ± | 51 | 1.0 | ± | 0.1 | 1.0 | ± | 0.1 | 17 | ± | 3 | 18 | ± | 3 |
| rib right 4 | 278 | ± | 53 | 276 | ± | 53 | 1.0 | ± | 0.1 | 1.0 | ± | 0.1 | 22 | ± | 4 | 22 | ± | 4 |
| rib right 5 | 277 | ± | 56 | 276 | ± | 56 | 1.0 | ± | 0.1 | 1.1 | ± | 0.1 | 26 | ± | 4 | 26 | ± | 4 |
| rib right 6 | 290 | ± | 57 | 291 | ± | 57 | 1.1 | ± | 0.2 | 1.1 | ± | 0.2 | 29 | ± | 4 | 29 | ± | 4 |
| rib right 7 | 295 | ± | 54 | 296 | ± | 51 | 1.1 | ± | 0.2 | 1.1 | ± | 0.2 | 30 | ± | 4 | 30 | ± | 5 |
| rib right 8 | 301 | ± | 54 | 302 | ± | 52 | 1.2 | ± | 0.2 | 1.2 | ± | 0.2 | 25 | ± | 4 | 25 | ± | 4 |
| rib right 9 | 306 | ± | 55 | 307 | ± | 54 | 1.1 | ± | 0.1 | 1.1 | ± | 0.2 | 23 | ± | 3 | 23 | ± | 3 |
| rib right 10 | 325 | ± | 56 | 324 | ± | 53 | 1.1 | ± | 0.1 | 1.1 | ± | 0.2 | 18 | ± | 3 | 18 | ± | 2 |
| rib right 11 | 347 | ± | 67 | 348 | ± | 66 | 1.1 | ± | 0.2 | 1.1 | ± | 0.2 | 12 | ± | 2 | 12 | ± | 2 |
| rib right 12 | 305 | ± | 64 | 305 | ± | 65 | 0.9 | ± | 0.1 | 1.0 | ± | 0.2 | 5 | ± | 3 | 6 | ± | 3 |
| rib right 13 | not present in the cohort | | | | | | | | | | | | | | | | | |
| sternum | 170 | ± | 30 | 170 | ± | 30 | 1.2 | ± | 0.2 | 1.2 | ± | 0.2 | 69 | ± | 9 | 70 | ± | 9 |
| vertebra C1 | 432 | ± | 49 | 431 | ± | 49 | 1.2 | ± | 0.1 | 1.3 | ± | 0.1 | 16 | ± | 2 | 16 | ± | 2 |
| vertebra C2 | 403 | ± | 50 | 403 | ± | 50 | 1.3 | ± | 0.1 | 1.3 | ± | 0.1 | 21 | ± | 3 | 21 | ± | 3 |
| vertebra C3 | 382 | ± | 54 | 385 | ± | 54 | 1.3 | ± | 0.1 | 1.3 | ± | 0.1 | 14 | ± | 2 | 14 | ± | 2 |
| vertebra C4 | 405 | ± | 53 | 406 | ± | 51 | 1.3 | ± | 0.1 | 1.3 | ± | 0.1 | 14 | ± | 2 | 14 | ± | 2 |
| vertebra C5 | 387 | ± | 50 | 391 | ± | 51 | 1.3 | ± | 0.1 | 1.3 | ± | 0.1 | 15 | ± | 2 | 15 | ± | 2 |
| vertebra C6 | 352 | ± | 46 | 353 | ± | 43 | 1.3 | ± | 0.1 | 1.3 | ± | 0.1 | 16 | ± | 2 | 16 | ± | 2 |
| vertebra C7 | 294 | ± | 38 | 295 | ± | 37 | 1.3 | ± | 0.2 | 1.3 | ± | 0.1 | 21 | ± | 2 | 21 | ± | 2 |
| vertebra T1 | 274 | ± | 35 | 273 | ± | 32 | 1.4 | ± | 0.2 | 1.4 | ± | 0.2 | 27 | ± | 3 | 27 | ± | 3 |
| vertebra T2 | 270 | ± | 35 | 269 | ± | 35 | 1.4 | ± | 0.2 | 1.4 | ± | 0.2 | 26 | ± | 3 | 27 | ± | 3 |
| vertebra T3 | 275 | ± | 37 | 274 | ± | 34 | 1.4 | ± | 0.2 | 1.5 | ± | 0.2 | 25 | ± | 3 | 25 | ± | 3 |
| vertebra T4 | 274 | ± | 38 | 274 | ± | 37 | 1.5 | ± | 0.2 | 1.5 | ± | 0.2 | 26 | ± | 3 | 26 | ± | 3 |
| vertebra T5 | 270 | ± | 36 | 270 | ± | 35 | 1.5 | ± | 0.2 | 1.5 | ± | 0.2 | 28 | ± | 3 | 28 | ± | 3 |
| vertebra T6 | 269 | ± | 35 | 268 | ± | 35 | 1.5 | ± | 0.2 | 1.5 | ± | 0.2 | 30 | ± | 4 | 30 | ± | 4 |
| vertebra T7 | 264 | ± | 33 | 263 | ± | 32 | 1.5 | ± | 0.2 | 1.5 | ± | 0.2 | 33 | ± | 5 | 33 | ± | 5 |
| vertebra T8 | 265 | ± | 37 | 260 | ± | 32 | 1.5 | ± | 0.3 | 1.6 | ± | 0.2 | 34 | ± | 8 | 35 | ± | 5 |
| vertebra T9 | 266 | ± | 36 | 264 | ± | 33 | 1.6 | ± | 0.2 | 1.6 | ± | 0.2 | 38 | ± | 5 | 38 | ± | 6 |
| vertebra T10 | 265 | ± | 45 | 259 | ± | 36 | 1.6 | ± | 0.2 | 1.6 | ± | 0.2 | 42 | ± | 8 | 42 | ± | 7 |
| vertebra T11 | 253 | ± | 36 | 249 | ± | 33 | 1.6 | ± | 0.2 | 1.6 | ± | 0.2 | 45 | ± | 6 | 45 | ± | 8 |
| vertebra T12 | 247 | ± | 34 | 242 | ± | 32 | 1.6 | ± | 0.2 | 1.6 | ± | 0.2 | 51 | ± | 7 | 51 | ± | 7 |
| vertebra L1 | 260 | ± | 36 | 257 | ± | 36 | 1.6 | ± | 0.2 | 1.6 | ± | 0.2 | 59 | ± | 10 | 58 | ± | 8 |
| vertebra L2 | 274 | ± | 39 | 272 | ± | 38 | 1.6 | ± | 0.2 | 1.5 | ± | 0.2 | 63 | ± | 9 | 63 | ± | 9 |
| vertebra L3 | 277 | ± | 40 | 275 | ± | 39 | 1.5 | ± | 0.2 | 1.5 | ± | 0.2 | 68 | ± | 10 | 69 | ± | 9 |
| vertebra L4 | 277 | ± | 38 | 278 | ± | 38 | 1.5 | ± | 0.2 | 1.5 | ± | 0.2 | 70 | ± | 10 | 69 | ± | 10 |
| vertebra L5 | 286 | ± | 36 | 285 | ± | 38 | 1.5 | ± | 0.3 | 1.5 | ± | 0.3 | 67 | ± | 10 | 67 | ± | 11 |
| vertebra L6 | 298 | ± | 110 | 390 | ± | 88 | 1.4 | ± | 0.3 | 1.4 | ± | 0.2 | 9 | ± | 17 | 9 | ± | 16 |
| hip left | 313 | ± | 41 | 313 | ± | 41 | 1.0 | ± | 0.1 | 1.0 | ± | 0.1 | 411 | ± | 53 | 416 | ± | 57 |
| hip right | 314 | ± | 41 | 314 | ± | 41 | 1.1 | ± | 0.1 | 1.2 | ± | 0.2 | 409 | ± | 53 | 407 | ± | 55 |
| sacrum | 207 | ± | 33 | 207 | ± | 33 | 1.2 | ± | 0.3 | 1.3 | ± | 0.2 | 231 | ± | 32 | 232 | ± | 33 |

**Table S4**. Selected reports of the normative values for CT (HU (mean ± STD)) and PET (SUV (mean ± STD)) for all 135 segmented regions, in addition to the CT-based volume estimates ([ml] (mean ± STD)), averaged across all 25 female healthy controls. FOV – field-of-view of the PET/CT system.

| **Organ** | **HU** | | | | | | **SUV** | | | | | | **Volume [ml]** | | | | | |
| --- | --- | --- | --- | --- | --- | --- | --- | --- | --- | --- | --- | --- | --- | --- | --- | --- | --- | --- |
|  | Test | | | Retest | | | Test | | | Retest | | | Test | | | Retest | | |
| skeletal muscle | 40 | ± | 9 | 40 | ± | 9 | 0.7 | ± | 0.0 | 0.6 | ± | 0.0 | 643 | ± | 128 | 656 | ± | 134 |
| subcutaneous fat | -78 | ± | 11 | -78 | ± | 12 | 0.4 | ± | 0.1 | 0.4 | ± | 0.1 | 758 | ± | 510 | 770 | ± | 541 |
| visceral fat | -63 | ± | 11 | -64 | ± | 11 | 0.9 | ± | 0.1 | 0.9 | ± | 0.2 | 200 | ± | 199 | 200 | ± | 202 |
| heart myocardium | 32 | ± | 7 | 32 | ± | 8 | 3.8 | ± | 2.6 | 4.2 | ± | 3.5 | 98 | ± | 15 | 98 | ± | 16 |
| heart atrium left | 41 | ± | 3 | 40 | ± | 3 | 2.0 | ± | 0.3 | 2.0 | ± | 0.3 | 52 | ± | 9 | 52 | ± | 10 |
| heart atrium right | 29 | ± | 9 | 27 | ± | 9 | 1.8 | ± | 0.2 | 1.8 | ± | 0.2 | 76 | ± | 14 | 78 | ± | 13 |
| heart ventricle left | 41 | ± | 3 | 40 | ± | 2 | 3.1 | ± | 1.6 | 3.1 | ± | 1.7 | 101 | ± | 15 | 100 | ± | 16 |
| heart ventricle right | 39 | ± | 4 | 38 | ± | 4 | 2.1 | ± | 0.6 | 2.1 | ± | 0.7 | 135 | ± | 20 | 137 | ± | 20 |
| aorta | 39 | ± | 2 | 39 | ± | 3 | 1.9 | ± | 0.2 | 1.9 | ± | 0.2 | 150 | ± | 50 | 148 | ± | 49 |
| iliac artery left | 41 | ± | 3 | 40 | ± | 3 | 1.6 | ± | 0.2 | 1.7 | ± | 0.4 | 10 | ± | 3 | 10 | ± | 3 |
| iliac artery right | 43 | ± | 3 | 42 | ± | 3 | 1.9 | ± | 0.2 | 1.8 | ± | 0.3 | 12 | ± | 4 | 13 | ± | 4 |
| iliac vena left | 41 | ± | 4 | 40 | ± | 3 | 1.9 | ± | 0.5 | 1.9 | ± | 0.5 | 27 | ± | 5 | 28 | ± | 6 |
| iliac vena right | 42 | ± | 3 | 42 | ± | 3 | 2.2 | ± | 0.6 | 2.0 | ± | 0.5 | 20 | ± | 6 | 21 | ± | 6 |
| inferior vena cava | 39 | ± | 3 | 39 | ± | 3 | 1.8 | ± | 0.2 | 1.8 | ± | 0.2 | 47 | ± | 16 | 46 | ± | 14 |
| portal splenic vein | 45 | ± | 9 | 45 | ± | 7 | 2.0 | ± | 0.3 | 1.8 | ± | 0.2 | 2 | ± | 3 | 3 | ± | 2 |
| pulmonary artery | 39 | ± | 3 | 40 | ± | 3 | 1.9 | ± | 0.2 | 1.9 | ± | 0.2 | 40 | ± | 9 | 40 | ± | 9 |
| colon | -198 | ± | 73 | -192 | ± | 102 | 1.8 | ± | 0.9 | 1.3 | ± | 0.5 | 777 | ± | 264 | 878 | ± | 313 |
| duodenum | -93 | ± | 106 | -99 | ± | 122 | 1.7 | ± | 0.3 | 1.7 | ± | 0.3 | 34 | ± | 18 | 35 | ± | 18 |
| esophagus | 9 | ± | 29 | 6 | ± | 33 | 1.8 | ± | 0.2 | 1.8 | ± | 0.2 | 24 | ± | 5 | 24 | ± | 6 |
| small bowel | -60 | ± | 74 | -98 | ± | 123 | 3.5 | ± | 1.1 | 2.7 | ± | 1.1 | 482 | ± | 199 | 529 | ± | 220 |
| autochthon left | 43 | ± | 9 | 43 | ± | 9 | 0.7 | ± | 0.1 | 0.7 | ± | 0.1 | 484 | ± | 102 | 485 | ± | 102 |
| autochthon right | 42 | ± | 10 | 42 | ± | 10 | 0.7 | ± | 0.1 | 0.7 | ± | 0.1 | 474 | ± | 98 | 476 | ± | 100 |
| gluteus maximus left | 41 | ± | 9 | 40 | ± | 9 | 0.6 | ± | 0.0 | 0.6 | ± | 0.1 | 557 | ± | 102 | 559 | ± | 108 |
| gluteus maximus right | 40 | ± | 9 | 40 | ± | 10 | 0.6 | ± | 0.0 | 0.6 | ± | 0.1 | 578 | ± | 107 | 582 | ± | 114 |
| gluteus medius left | 45 | ± | 5 | 44 | ± | 5 | 0.7 | ± | 0.1 | 0.6 | ± | 0.1 | 229 | ± | 38 | 229 | ± | 40 |
| gluteus medius right | 46 | ± | 5 | 45 | ± | 5 | 0.7 | ± | 0.1 | 0.7 | ± | 0.1 | 232 | ± | 42 | 232 | ± | 42 |
| gluteus minimus left | 45 | ± | 9 | 45 | ± | 9 | 0.7 | ± | 0.2 | 0.7 | ± | 0.1 | 54 | ± | 9 | 54 | ± | 9 |
| gluteus minimus right | 44 | ± | 9 | 44 | ± | 9 | 0.8 | ± | 0.1 | 0.7 | ± | 0.1 | 59 | ± | 11 | 60 | ± | 11 |
| iliopsoas left | 54 | ± | 3 | 54 | ± | 3 | 0.7 | ± | 0.1 | 0.7 | ± | 0.1 | 289 | ± | 69 | 292 | ± | 69 |
| iliopsoas right | 52 | ± | 3 | 52 | ± | 3 | 0.8 | ± | 0.1 | 0.8 | ± | 0.1 | 280 | ± | 66 | 284 | ± | 66 |
| adrenal gland left | 26 | ± | 12 | 27 | ± | 15 | 1.9 | ± | 0.2 | 1.8 | ± | 0.2 | 1 | ± | 1 | 1 | ± | 1 |
| adrenal gland right | 16 | ± | 13 | 14 | ± | 11 | 1.9 | ± | 0.3 | 1.8 | ± | 0.3 | 1 | ± | 1 | 1 | ± | 1 |
| bladder | 20 | ± | 11 | 17 | ± | 10 | 29.0 | ± | 21.8 | 37.0 | ± | 32.1 | 68 | ± | 54 | 78 | ± | 42 |
| brain | 35 | ± | 2 | 35 | ± | 1 | 6.9 | ± | 0.9 | 6.9 | ± | 0.7 | 1299 | ± | 120 | 1299 | ± | 121 |
| gallbladder | 18 | ± | 5 | 18 | ± | 5 | 1.1 | ± | 0.4 | 1.1 | ± | 0.3 | 18 | ± | 9 | 20 | ± | 9 |
| kidney left | 28 | ± | 4 | 27 | ± | 5 | 2.9 | ± | 0.4 | 3.2 | ± | 0.8 | 105 | ± | 37 | 101 | ± | 38 |
| kidney right | 29 | ± | 3 | 29 | ± | 4 | 3.3 | ± | 0.8 | 3.5 | ± | 1.0 | 110 | ± | 34 | 106 | ± | 35 |
| liver | 55 | ± | 4 | 55 | ± | 4 | 2.3 | ± | 0.3 | 2.3 | ± | 0.3 | 1412 | ± | 279 | 1447 | ± | 250 |
| lung lower lobe left | -650 | ± | 68 | -640 | ± | 72 | 0.7 | ± | 0.1 | 0.8 | ± | 0.1 | 579 | ± | 127 | 563 | ± | 126 |
| lung lower lobe right | -668 | ± | 60 | -663 | ± | 65 | 0.7 | ± | 0.2 | 0.7 | ± | 0.2 | 648 | ± | 145 | 638 | ± | 146 |
| lung middle lobe right | -773 | ± | 42 | -771 | ± | 43 | 0.5 | ± | 0.1 | 0.5 | ± | 0.1 | 285 | ± | 64 | 284 | ± | 63 |
| lung upper lobe left | -736 | ± | 49 | -732 | ± | 54 | 0.6 | ± | 0.1 | 0.6 | ± | 0.1 | 695 | ± | 201 | 689 | ± | 215 |
| lung upper lobe right | -744 | ± | 45 | -740 | ± | 50 | 0.5 | ± | 0.1 | 0.5 | ± | 0.1 | 561 | ± | 137 | 559 | ± | 150 |
| pancreas | 45 | ± | 4 | 44 | ± | 4 | 1.6 | ± | 0.2 | 1.6 | ± | 0.2 | 66 | ± | 19 | 68 | ± | 19 |
| spleen | 40 | ± | 4 | 40 | ± | 5 | 1.7 | ± | 0.2 | 1.8 | ± | 0.2 | 165 | ± | 62 | 161 | ± | 61 |
| stomach | -170 | ± | 129 | -130 | ± | 125 | 1.5 | ± | 0.3 | 1.6 | ± | 0.3 | 222 | ± | 107 | 192 | ± | 62 |
| thyroid left | 74 | ± | 10 | 72 | ± | 11 | 1.3 | ± | 0.2 | 1.3 | ± | 0.4 | 5 | ± | 1 | 5 | ± | 1 |
| thyroid right | 75 | ± | 10 | 73 | ± | 10 | 1.3 | ± | 0.2 | 1.4 | ± | 0.4 | 5 | ± | 1 | 5 | ± | 1 |
| trachea | -838 | ± | 17 | -834 | ± | 19 | 0.8 | ± | 0.1 | 0.8 | ± | 0.1 | 25 | ± | 6 | 25 | ± | 6 |
| carpal left | 251 | ± | 51 | 257 | ± | 82 | 0.6 | ± | 0.2 | 0.6 | ± | 0.2 | 17 | ± | 9 | 18 | ± | 8 |
| carpal right | 238 | ± | 39 | 253 | ± | 28 | 0.6 | ± | 0.2 | 0.7 | ± | 0.2 | 16 | ± | 7 | 18 | ± | 4 |
| clavicle left | 371 | ± | 59 | 373 | ± | 59 | 0.9 | ± | 0.2 | 0.8 | ± | 0.1 | 26 | ± | 5 | 26 | ± | 5 |
| clavicle right | 374 | ± | 55 | 375 | ± | 56 | 0.9 | ± | 0.2 | 0.8 | ± | 0.1 | 26 | ± | 6 | 26 | ± | 6 |
| femur left | 432 | ± | 46 | 429 | ± | 45 | 0.6 | ± | 0.2 | 0.6 | ± | 0.2 | 242 | ± | 27 | 241 | ± | 27 |
| femur right | 429 | ± | 47 | 427 | ± | 45 | 0.7 | ± | 0.2 | 0.6 | ± | 0.2 | 242 | ± | 25 | 240 | ± | 25 |
| fibula left | not in FOV | | | | | | | | | | | | | | | | | |
| fibula right | not in FOV | | | | | | | | | | | | | | | | | |
| fingers left | 317 | ± | 42 | 319 | ± | 44 | 0.6 | ± | 0.2 | 0.6 | ± | 0.2 | 8 | ± | 6 | 8 | ± | 5 |
| fingers right | 320 | ± | 42 | 321 | ± | 54 | 0.7 | ± | 0.2 | 0.7 | ± | 0.2 | 10 | ± | 4 | 10 | ± | 4 |
| humerus left | 349 | ± | 35 | 340 | ± | 36 | 0.5 | ± | 0.1 | 0.5 | ± | 0.1 | 163 | ± | 29 | 162 | ± | 30 |
| humerus right | 353 | ± | 35 | 352 | ± | 34 | 0.5 | ± | 0.1 | 0.5 | ± | 0.1 | 167 | ± | 29 | 166 | ± | 30 |
| metacarpal left | 300 | ± | 57 | 307 | ± | 39 | 0.6 | ± | 0.2 | 0.6 | ± | 0.2 | 21 | ± | 13 | 22 | ± | 10 |
| metacarpal right | 299 | ± | 52 | 319 | ± | 64 | 0.6 | ± | 0.2 | 0.7 | ± | 0.2 | 25 | ± | 10 | 28 | ± | 8 |
| metatarsal left | not in FOV | | | | | | | | | | | | | | | | | |
| metatarsal right | not in FOV | | | | | | | | | | | | | | | | | |
| patella left | not in FOV | | | | | | | | | | | | | | | | | |
| patella right | not in FOV | | | | | | | | | | | | | | | | | |
| radius left | 393 | ± | 58 | 396 | ± | 52 | 0.6 | ± | 0.1 | 0.6 | ± | 0.1 | 37 | ± | 12 | 39 | ± | 14 |
| radius right | 404 | ± | 58 | 399 | ± | 57 | 0.5 | ± | 0.1 | 0.5 | ± | 0.1 | 40 | ± | 11 | 41 | ± | 10 |
| scapula left | 317 | ± | 42 | 317 | ± | 42 | 0.8 | ± | 0.1 | 0.8 | ± | 0.1 | 87 | ± | 18 | 87 | ± | 17 |
| scapula right | 323 | ± | 41 | 323 | ± | 41 | 0.9 | ± | 0.1 | 0.8 | ± | 0.1 | 89 | ± | 17 | 89 | ± | 18 |
| skull | 584 | ± | 52 | 583 | ± | 53 | 1.8 | ± | 0.4 | 1.8 | ± | 0.3 | 751 | ± | 98 | 751 | ± | 97 |
| tarsal left | not in FOV | | | | | | | | | | | | | | | | | |
| tarsal right | not in FOV | | | | | | | | | | | | | | | | | |
| tibia left | not in FOV | | | | | | | | | | | | | | | | | |
| tibia right | not in FOV | | | | | | | | | | | | | | | | | |
| toes left | not in FOV | | | | | | | | | | | | | | | | | |
| toes right | not in FOV | | | | | | | | | | | | | | | | | |
| ulna left | 364 | ± | 56 | 351 | ± | 56 | 0.5 | ± | 0.1 | 0.5 | ± | 0.1 | 46 | ± | 14 | 48 | ± | 13 |
| ulna right | 367 | ± | 64 | 362 | ± | 59 | 0.5 | ± | 0.1 | 0.5 | ± | 0.1 | 51 | ± | 12 | 49 | ± | 12 |
| rib left 1 | 297 | ± | 55 | 295 | ± | 56 | 1.0 | ± | 0.2 | 0.9 | ± | 0.2 | 8 | ± | 2 | 8 | ± | 2 |
| rib left 2 | 290 | ± | 65 | 288 | ± | 65 | 0.9 | ± | 0.1 | 0.9 | ± | 0.1 | 11 | ± | 2 | 11 | ± | 2 |
| rib left 3 | 297 | ± | 72 | 298 | ± | 74 | 0.9 | ± | 0.1 | 0.9 | ± | 0.1 | 13 | ± | 3 | 13 | ± | 3 |
| rib left 4 | 305 | ± | 68 | 306 | ± | 70 | 1.0 | ± | 0.1 | 0.9 | ± | 0.1 | 16 | ± | 3 | 16 | ± | 3 |
| rib left 5 | 307 | ± | 69 | 308 | ± | 71 | 1.0 | ± | 0.1 | 1.0 | ± | 0.1 | 18 | ± | 3 | 18 | ± | 3 |
| rib left 6 | 318 | ± | 61 | 318 | ± | 61 | 1.0 | ± | 0.1 | 1.0 | ± | 0.1 | 21 | ± | 3 | 21 | ± | 4 |
| rib left 7 | 327 | ± | 62 | 327 | ± | 64 | 1.0 | ± | 0.1 | 1.0 | ± | 0.1 | 22 | ± | 3 | 22 | ± | 3 |
| rib left 8 | 331 | ± | 66 | 330 | ± | 66 | 1.0 | ± | 0.1 | 1.0 | ± | 0.1 | 18 | ± | 3 | 18 | ± | 3 |
| rib left 9 | 324 | ± | 66 | 323 | ± | 66 | 1.1 | ± | 0.1 | 1.0 | ± | 0.1 | 17 | ± | 3 | 17 | ± | 3 |
| rib left 10 | 337 | ± | 62 | 337 | ± | 61 | 1.1 | ± | 0.1 | 1.0 | ± | 0.1 | 14 | ± | 3 | 13 | ± | 2 |
| rib left 11 | 351 | ± | 70 | 351 | ± | 70 | 1.0 | ± | 0.1 | 1.0 | ± | 0.1 | 9 | ± | 2 | 9 | ± | 2 |
| rib left 12 | 323 | ± | 68 | 326 | ± | 67 | 1.2 | ± | 0.2 | 1.1 | ± | 0.2 | 4 | ± | 2 | 4 | ± | 2 |
| rib left 13 | not present in the cohort | | | | | | | | | | | | | | | | | |
| rib right 1 | 310 | ± | 54 | 304 | ± | 55 | 1.0 | ± | 0.2 | 1.0 | ± | 0.2 | 8 | ± | 2 | 8 | ± | 2 |
| rib right 2 | 304 | ± | 67 | 303 | ± | 67 | 0.9 | ± | 0.1 | 0.9 | ± | 0.1 | 11 | ± | 2 | 11 | ± | 2 |
| rib right 3 | 301 | ± | 75 | 303 | ± | 74 | 0.9 | ± | 0.1 | 0.9 | ± | 0.1 | 12 | ± | 3 | 12 | ± | 3 |
| rib right 4 | 310 | ± | 73 | 312 | ± | 74 | 0.9 | ± | 0.1 | 0.9 | ± | 0.1 | 16 | ± | 3 | 16 | ± | 3 |
| rib right 5 | 311 | ± | 71 | 313 | ± | 72 | 1.0 | ± | 0.1 | 1.0 | ± | 0.1 | 18 | ± | 4 | 18 | ± | 4 |
| rib right 6 | 323 | ± | 67 | 323 | ± | 68 | 1.1 | ± | 0.2 | 1.1 | ± | 0.2 | 21 | ± | 4 | 21 | ± | 4 |
| rib right 7 | 331 | ± | 62 | 332 | ± | 62 | 1.1 | ± | 0.2 | 1.1 | ± | 0.2 | 21 | ± | 4 | 21 | ± | 4 |
| rib right 8 | 336 | ± | 64 | 336 | ± | 64 | 1.2 | ± | 0.1 | 1.1 | ± | 0.2 | 18 | ± | 3 | 18 | ± | 3 |
| rib right 9 | 331 | ± | 65 | 331 | ± | 65 | 1.2 | ± | 0.1 | 1.2 | ± | 0.1 | 16 | ± | 3 | 16 | ± | 3 |
| rib right 10 | 350 | ± | 63 | 351 | ± | 62 | 1.2 | ± | 0.2 | 1.2 | ± | 0.1 | 13 | ± | 3 | 13 | ± | 2 |
| rib right 11 | 361 | ± | 67 | 362 | ± | 67 | 1.2 | ± | 0.2 | 1.2 | ± | 0.2 | 8 | ± | 2 | 8 | ± | 2 |
| rib right 12 | 335 | ± | 64 | 335 | ± | 64 | 1.1 | ± | 0.2 | 1.1 | ± | 0.2 | 4 | ± | 2 | 3 | ± | 2 |
| rib right 13 | not present in the cohort | | | | | | | | | | | | | | | | | |
| sternum | 147 | ± | 39 | 149 | ± | 40 | 1.2 | ± | 0.3 | 1.2 | ± | 0.3 | 51 | ± | 12 | 51 | ± | 12 |
| vertebra C1 | 408 | ± | 50 | 408 | ± | 50 | 1.3 | ± | 0.1 | 1.3 | ± | 0.1 | 14 | ± | 2 | 14 | ± | 2 |
| vertebra C2 | 387 | ± | 58 | 386 | ± | 58 | 1.4 | ± | 0.1 | 1.3 | ± | 0.1 | 17 | ± | 2 | 17 | ± | 2 |
| vertebra C3 | 379 | ± | 63 | 377 | ± | 63 | 1.3 | ± | 0.1 | 1.3 | ± | 0.1 | 12 | ± | 2 | 12 | ± | 2 |
| vertebra C4 | 405 | ± | 61 | 404 | ± | 61 | 1.3 | ± | 0.1 | 1.3 | ± | 0.1 | 11 | ± | 2 | 11 | ± | 2 |
| vertebra C5 | 392 | ± | 62 | 391 | ± | 58 | 1.3 | ± | 0.1 | 1.3 | ± | 0.1 | 12 | ± | 2 | 12 | ± | 2 |
| vertebra C6 | 353 | ± | 54 | 353 | ± | 54 | 1.3 | ± | 0.1 | 1.3 | ± | 0.1 | 13 | ± | 2 | 13 | ± | 2 |
| vertebra C7 | 305 | ± | 44 | 303 | ± | 44 | 1.3 | ± | 0.1 | 1.3 | ± | 0.1 | 17 | ± | 3 | 17 | ± | 3 |
| vertebra T1 | 277 | ± | 40 | 276 | ± | 40 | 1.4 | ± | 0.1 | 1.3 | ± | 0.2 | 21 | ± | 4 | 21 | ± | 4 |
| vertebra T2 | 271 | ± | 40 | 271 | ± | 42 | 1.4 | ± | 0.2 | 1.4 | ± | 0.2 | 21 | ± | 3 | 21 | ± | 3 |
| vertebra T3 | 271 | ± | 42 | 270 | ± | 41 | 1.5 | ± | 0.2 | 1.4 | ± | 0.2 | 20 | ± | 3 | 20 | ± | 3 |
| vertebra T4 | 264 | ± | 39 | 264 | ± | 39 | 1.5 | ± | 0.2 | 1.4 | ± | 0.2 | 21 | ± | 3 | 21 | ± | 4 |
| vertebra T5 | 262 | ± | 40 | 261 | ± | 40 | 1.5 | ± | 0.2 | 1.4 | ± | 0.2 | 22 | ± | 4 | 22 | ± | 4 |
| vertebra T6 | 261 | ± | 42 | 260 | ± | 41 | 1.5 | ± | 0.2 | 1.5 | ± | 0.2 | 24 | ± | 4 | 24 | ± | 4 |
| vertebra T7 | 257 | ± | 40 | 256 | ± | 38 | 1.5 | ± | 0.2 | 1.5 | ± | 0.2 | 26 | ± | 4 | 26 | ± | 4 |
| vertebra T8 | 254 | ± | 43 | 252 | ± | 41 | 1.5 | ± | 0.2 | 1.5 | ± | 0.2 | 28 | ± | 5 | 28 | ± | 5 |
| vertebra T9 | 255 | ± | 44 | 255 | ± | 43 | 1.5 | ± | 0.2 | 1.5 | ± | 0.2 | 30 | ± | 5 | 30 | ± | 5 |
| vertebra T10 | 259 | ± | 45 | 258 | ± | 45 | 1.6 | ± | 0.2 | 1.5 | ± | 0.2 | 33 | ± | 5 | 33 | ± | 6 |
| vertebra T11 | 251 | ± | 45 | 250 | ± | 45 | 1.6 | ± | 0.2 | 1.5 | ± | 0.2 | 36 | ± | 6 | 36 | ± | 6 |
| vertebra T12 | 252 | ± | 49 | 251 | ± | 47 | 1.6 | ± | 0.2 | 1.5 | ± | 0.2 | 40 | ± | 7 | 40 | ± | 7 |
| vertebra L1 | 265 | ± | 49 | 266 | ± | 48 | 1.5 | ± | 0.2 | 1.5 | ± | 0.2 | 45 | ± | 7 | 46 | ± | 7 |
| vertebra L2 | 281 | ± | 49 | 280 | ± | 51 | 1.5 | ± | 0.2 | 1.4 | ± | 0.2 | 50 | ± | 8 | 50 | ± | 7 |
| vertebra L3 | 280 | ± | 50 | 280 | ± | 51 | 1.5 | ± | 0.2 | 1.4 | ± | 0.2 | 55 | ± | 8 | 56 | ± | 8 |
| vertebra L4 | 281 | ± | 49 | 280 | ± | 50 | 1.5 | ± | 0.2 | 1.4 | ± | 0.2 | 58 | ± | 8 | 57 | ± | 8 |
| vertebra L5 | 278 | ± | 48 | 279 | ± | 48 | 1.5 | ± | 0.2 | 1.4 | ± | 0.2 | 57 | ± | 8 | 57 | ± | 8 |
| vertebra L6 | 316 | ± | 149 | 310 | ± | 137 | 1.6 | ± | 0.8 | 1.1 | ± | 0.2 | 1 | ± | 1 | 0 | ± | 0 |
| hip left | 306 | ± | 54 | 304 | ± | 54 | 1.1 | ± | 0.2 | 1.1 | ± | 0.2 | 330 | ± | 50 | 330 | ± | 50 |
| hip right | 308 | ± | 55 | 308 | ± | 56 | 1.3 | ± | 0.2 | 1.2 | ± | 0.2 | 327 | ± | 50 | 326 | ± | 51 |
| sacrum | 183 | ± | 41 | 183 | ± | 42 | 1.3 | ± | 0.3 | 1.2 | ± | 0.2 | 215 | ± | 28 | 215 | ± | 29 |
